# Water Promotes Melting of a Metal–Organic Framework

**DOI:** 10.1021/acs.chemmater.3c02873

**Published:** 2024-03-06

**Authors:** Søren S. Sørensen, Anders K. R. Christensen, Elena A. Bouros-Bandrabur, Emil S. Andersen, Heidi F. Christiansen, Sofie Lang, Fengming Cao, M. Faizal Ussama Jalaludeen, Johan F. S. Christensen, Wessel M. W. Winters, Bettina P. Andersen, Anders B. Nielsen, Niels Chr. Nielsen, Dorthe B. Ravnsbæk, Peter K. Kristensen, Yuanzheng Yue, Morten M. Smedskjaer

**Affiliations:** † Department of Chemistry and Bioscience, Aalborg University, Aalborg DK-9220, Denmark; ‡ Department of Chemistry, Aarhus University, Aarhus DK-8000, Denmark; § Interdisciplinary Nanoscience Center (iNANO), Aarhus University, Aarhus DK-8000, Denmark; ∥ Department of Materials and Production, Aalborg University, Aalborg DK-9220, Denmark

## Abstract

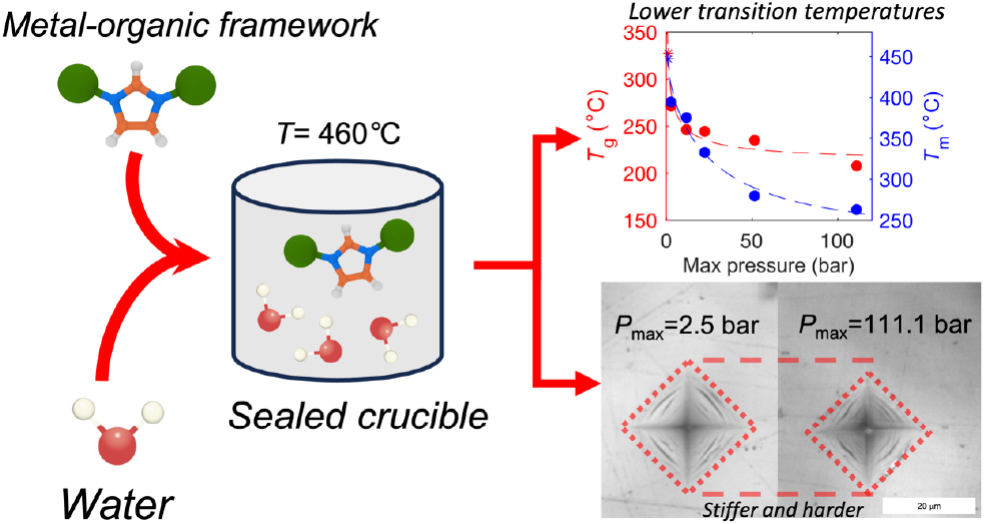

Water is one of the most reactive and abundant molecules on Earth, and it is thus crucial to understand its reactivity with various material families. One of the big unknown questions is how water in liquid and vapor forms impact the fast-emerging class of metal–organic frameworks (MOFs). Here, we discover that high-pressure water vapor drastically modifies the structure and hence the dynamic, thermodynamic, and mechanical properties of MOF glasses. In detail, we find that an archetypical MOF (ZIF-62) is extremely sensitive to heat treatments performed at 460 °C and water vapor pressures up to ∼110 bar. Both the melting and glass transition temperatures decrease remarkably (by >100 °C), and simultaneously, hardness and Young’s modulus increase by up to 100% under very mild treatment conditions (<20 bar of hydrothermal pressure). Structural analyses suggest water to partially coordinate to Zn in the form of a hydroxide ion by replacing a bridging imidazolate-based linker. The work provides insight into the role of hot-compressed water in influencing the structure and properties of MOF glasses and opens a new route for systematically changing the thermodynamics and kinetics of MOF liquids and thus altering the thermal and mechanical properties of the resulting MOF glasses.

## Introduction

Metal–organic frameworks (MOFs) are composed of metal-ion nodes connected by organic-linkers.^1^ Over the past decade, a variety of amorphous MOFs made by mechanochemical synthesis have been reported, and in 2015, the first melt-quenched MOF glasses were discovered.^2,3^ The most studied MOF glass family is zeolitic imidazolate frameworks (ZIFs)^4^ since they exhibit high glass forming ability due to high *T*_g_/*T*_m_ ratio (>0.8), where *T*_g_ and *T*_m_ are the glass transition temperature and the melting point of ZIFs, respectively.^2,3,5^ ZIFs consist of metal-ion nodes (Zn, Co, Fe) connected with imidazolate-type linkers through metal–nitrogen coordination bonds. ZIF glasses show great promise as materials for membranes,^6,7^ batteries,^8^ and as stabilizing agents for halide perovskite light-emitting diodes.^9^ However, one of the most significant obstacles toward such applications and sample scale-up is the proximity of the melting (*T*_m_) and decomposition (*T*_d_) temperatures.^10^ To this end, the effect of water is crucial to explore considering its abundance and reactivity, for example, it is known to lower *T*_m_ and *T*_g_ of oxides. Indeed, some progress has been made through combination of hybrid systems with liquid water (and some ionic liquids),^11−13^ but there is the lack of routes for creating defect-free materials at reduced temperatures^14^ and for continuous property alteration. The latter is a hallmark of traditional glass families (oxides, chalcogenides, metallic)^15^ but has not yet been achieved in hybrid glasses.

Coordination chemistry is featured by the interaction between electron-donating ligands and metal ions, but the chemistry of the ligands varies significantly and determines the final bond strength and stability. This has been the basis for lowering *T*_m_ and *T*_g_ in a cobalt bis-acetamide hybrid glass using water as a replacement ligand^12^ as well as in ZIF-8/62/76 by incorporating ionic liquids.^11,16^ Other approaches include changing the ligand chemistry in ZIFs, e.g., the imidazolate to benzimidazolate ligand ratio^17,18^ or the introduction of cyano-substituted ligands.^19^ The latter approach resulted in a very low melting point of ZIF-4 (∼500 K).^19^ Moreover, the weak coordination bonds in ZIFs are sensitive to even moderate pressures (from tens of MPa to few GPa), resulting in changes in transition temperatures (*T*_m_ changes^20^ by ∼70 K and *T*_g_ changes^21^ by ∼40 K). As such, while several methods exist for decreasing *T*_m_ and *T*_g_, approaches for a unified and continuous decrease of *T*_m_ and *T*_g_ are lacking. Inspired by the previous work on both water and high-pressure sensitivity in MOF glasses,^12,20,21^ and further motivated by the importance of understanding water–MOF interactions, we here reveal that water-assisted compression and modification of the MOF structure through hydrothermal treatment leads to unprecedently low *T*_m_ and *T*_g_ values of an archetypical ZIF glass, namely, ZIF-62 (ZnIm_1.75_bIm_0.25_, where Im is imidazolate and bIm is benzimidazolate). The treatment is performed by heating sealed high-pressure crucibles containing water to 460 °C, which was chosen considering the ambient pressure melting point (∼450 °C) and the maximum temperature of the used high-pressure crucibles (500 °C). Notably, the treatment simultaneously makes the glass stiffer and harder, unlike the case in oxide glasses, where lower *T*_m_ and *T*_g_ values are almost always associated with softer glasses.

## Methods

### Synthesis

The synthetic procedure to prepare crystalline ZIF-62 follows that of previous reports.^22,23^ Specifically, zinc nitrate hexahydrate (3.79 g, 12.74 mmol), imidazole (5.69 g, 83.58 mmol), and benzimidazole (1.60 g, 13.54 mmol) were dissolved in 75 mL of *N*,*N*-dimethylformamide (DMF). The mixture was then stirred until all solids were dissolved. Now, the solution was transferred to a Teflon-lined autoclave where the solution was heated in an oven for 72 h at 120 °C before cooling to room temperature. The formed ZIF-62 was transferred to a 50 mL centrifuge vial, and 30 mL fresh DMF was added. This mixture was then centrifuged for 5 min at 3000 rpm. This procedure was repeated twice, but the last one used 30 mL dichloromethane instead of DMF. Finally, some ZIF-62 was dried at ∼350 °C under an argon atmosphere to remove residual DMF from the pores of the ZIF-62 structure. As shown in Figure S1, the experimental composition of the as-synthesized ZIF-62 crystal was found to be Zn(imidazolate)_1.724_(benzimidazolate)_0.276_ based on ^1^H NMR spectroscopy analysis of a digested sample. This is close to the nominal composition of Zn(imidazolate)_1.75_(benzimidazolate)_0.25_. A similar composition is found for the dried ZIF-62 crystal (Figure S2).

### Glass Formation and Calorimetry

All differential scanning calorimetry (DSC) measurements were performed by using a Netzsch STA F449 F3 instrument equipped with liquid N_2_ cooling or a Netzsch STA F449 F5 instrument. Noncompressed samples were measured in Netzsch cold-welded aluminum crucibles. The other sample measurements with added water were performed using 100 μL BFT 94 crucibles (Bächler Feintech AG). Specifically, these are steel crucibles sealed using a steel screw cap pressing onto a gold lid (with a torque of 3.7 N m) ensuring tightness up to ∼270 bar of pressure. Demineralized water was added using an adjustable Finnpipette (0.1–1.0 μL), and water masses were confirmed by subsequent measurements of the total mass of sample and water in the crucible. Melting temperatures (*T*_m_) were determined as offset values of the endothermal melting peak, while glass transition temperatures (*T*_g_) were determined as the onset temperature. Similarly to the experiments with water, experiments with ethanol and *n*-hexane were performed to mimic the experiments with the highest water content. Here, the solvents were added to the pressure tight crucibles (now in a gold coated version to avoid any possible reactions between the solvents and the container) in a similar molar ratio and with a similar amount of ZIF sample mass as for the water experiments of highest water content. This was done to ensure similar maximum pressures at all temperatures above the evaporation point of the solvent.

### Estimation of Pressure Inside High Pressure Crucibles

Before glass formation, each 100 μL high-pressure crucible was loaded with approximately 10 mg of ZIF-62. Varying amounts of demineralized water were then added (from 0 to approximately 3 μL). Due to the encapsulation of the sample and water, the boiling point of water (and thus the temperature at which all water is transformed to gas) was significantly above that under atmospheric conditions. To estimate whether all water evaporated inside the crucible, we used the Antoine equation to estimate the vapor pressure of water (*p*),
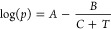
1where *A*, *B*, and *C* are empirical constants and *T* is temperature. By plotting the vapor pressure as well as the estimated gas pressure using the ideal gas equation, we estimate the boiling point from the interception between the pressure of the ideal gas equation and that from eq 1. As shown in Figure S3, all samples feature boiling points below 300 °C. Since all samples are subjected to heating above these boiling points of water, an estimation of the pressure inside the crucible relies on the gas pressure. To this end, we used the ideal gas equation to determine the pressure at the maximum temperature (Figure S3).

### Indentation-Based Mechanical Properties

Prior to indentation experiments, the ZIF-62 glass samples were ground by using SiC grinding papers to obtain a level sample surface. The surfaces were then polished using a water-free 3 μm diamond suspension. A series of at least eight Vickers indentations were made on each sample using a Nanovea CB500 instrumented indentation apparatus. A peak load of 0.2 N and a hold time of 15 s at peak load were used. Both the loading and unloading rates were 0.8 N/min. During indentation experiments, both the load (*P*) and displacement (*h*) were continuously measured using the instrument’s load cell and optical noncontact depth sensor, respectively, to obtain load–displacement (*P–h*) curves.

The obtained load–displacement curves were then analyzed using the Oliver*–*Pharr method.^24,25^ For each load–displacement curve, the function *P*(*h*) = *B*(*h* – *h*_f_)*^k^* was fitted to the unloading data from 0.195 to 0.040 N by using a least-squares procedure to optimize the three fitting parameters *h*_f_, *k*, and *B*, where *h*_f_ corresponds to the displacement at zero load during unloading and the value of *k* is related to the indenter geometry. Using *P*_max_ = 0.2 N and the fitted parameters, *h*_max_ was calculated as *h*_max_ = *h*_f_ + (*P*_max_/*B*)^1/*k*^. The stiffness *S* (i.e., the slope of the unloading curve at *h*_max_) was then calculated as *S* = *kB*(*h*_max_ – *h*_f_)*^k^*^–1^. The contact depth *h*_c_ was estimated as *h*_c_ = *h*_max_*–* 0.75*P*_max_/*S* and used to estimate the indentation contact area projected to the surface *A*_c_ = [2tan(136°/2)*h*_c_]^2^. Then, the reduced modulus was calculated as *E*_r_ = 0.5062π^2^*S*/*A*_c_^0.5^. Lastly, the indentation contact area was estimated as *A*_i_ = [4tan(136°/2)/cos(136°/2)]*h*_c_^2^ and used to calculate the sample Vickers hardness as *H* = *P*_max_/*A*_i_.

We also compared the obtained load–displacement-based Vickers hardness from the glass produced without hydrothermal pressure (and thus only at a maximum of 2.5 bar of Ar gas, value of *H*_V_ = 0.56 GPa) with a previous report of *H*_V_ based on microscopic analysis of the indents (*H*_V_ = 0.525 GPa),^21,26^ finding very good agreement.

### Powder X-Ray Diffraction Analysis

Samples were measured on a zero-background plate made of monocrystalline silicon. Measurements were conducted using a Panalytical Empyrean X-ray diffractometer equipped with a Cu source (λ_Kα_ = 1.5406 Å).

### Pair Distribution Function Analysis

ZIF-62 glass samples were mounted in 0.0342” (∼0.86 mm) Kapton capillaries, and total X-ray scattering data were collected using a STOE STADI P laboratory diffractometer. The instrument is equipped with four MYTHEN 4K detectors and a Ag Kα radiation source (λ = 0.559407 Å). The data were collected using moving mode covering a 2θ range of 2 to 118° and with a total measurement time of 24 h. The total scattering data were subsequently reduced, to obtain *S*(*Q*) and Fourier transformed to the pair distribution function (PDF) *G*(*r*) using the PDFgetX3.^27^ A *Q*_max_ of 18.4 Å^–1^ and an *r*_poly_ value of 1.1 Å were used.

### Fourier Transform Infrared Spectroscopy (FTIR)

FTIR spectra were recorded by using an attenuated total reflection setup on a Bruker Tensor II spectrometer. Crystalline diamond was used as the attenuation crystal. All samples were measured under ambient conditions in the 500–4000 cm^–1^ frequency range.

### Solid State ^67^Zn NMR

Solid-state NMR analyses were carried out on a 22.3 T Bruker Avance III HD narrow-bore (950 MHz for ^1^H) spectrometer equipped with a 4 mm HX double-resonance probe. The ^67^Zn (*I* = 5/2) NMR data were recorded using magic-angle spinning (MAS) with a spinning frequency of 15 kHz and a Hahn-echo pulse sequence using 15.8 kHz rf field strength with a total echo-time of one rotor period (pulse times of 4 and 8 ms were used for the initial π/2 pulse and the echo π pulse, adjusted to compensate for rf pulse effects influenced by quadrupolar coupling). The spectrum for the sample prepared at 1 bar was acquired for a mass of 92 mg and employed 1,447,978 scans. The spectrum for the sample prepared at 11 bar was acquired for a mass of 99 mg and employed 1,810,200 scans. Both spectra used a repetition delay of 0.1 s. In addition, an empty rotor experiment was recorded under the same experimental conditions, and the spectrum was subtracted from the sample spectra (see Figure S4) to remove effects from the ^67^Zn NMR background. Isotropic chemical shifts are relative to those in an aqueous 1.0 M solution of Zn(NO_3_)_2_. Simulation of the experimental spectra was performed using the open-source SIMPSON software^28^ by adding a series of 231 powder spectra (each representing 4180 crystallite orientations selected using the Zaremba–Conroy–Wolfsberg method)^29−31^ with quadrupolar coupling (*C*_Q_) and asymmetry (*η*) parameters distributed in a grid equidistantly from 1 to 11 MHz (21 values) and 0 to 1 (11 values), respectively, and weighted according to the Czjzek model for disordered solids^32^ with <*C*^2^_Q*η*_> = 6.5 MHz (see plot of weighting values in Figure S5; the simulated spectra were apodized with 350 Hz Lorentzian linebroadening). Apart from a minor isotropic shift (7.5 ppm), all NMR interaction parameters match earlier report by Madsen et al. for ZIF-62 glass.^33^

### Solid State ^1^H NMR

Solid-state ^1^H NMR experiments were carried out on a 22.3 T Bruker Avance III HD narrow-bore (950 MHz for ^1^H) spectrometer equipped with a 1.9 mm ^1^H–^13^C–^15^N–^2^H probe. The ^1^H NMR data were recorded by using magic-angle spinning with a spinning frequency of 35 kHz. Both spectra used a repetition delay of 3.0 s. Isotropic chemical shifts are relative to ^1^H for adamantane at 1.82 ppm.

### Liquid State ^1^H NMR

Liquid-state NMR analyses were performed on a Bruker Avance III 600 MHz (14.1 T) spectrometer. Solid samples (5–10 mg) were digested in 400 μL of 1:5, and DCl (35 wt % in D_2_O, ≥ 99% *d*): DMSO–*d*_6_ (99.8% D) solutions were used prior to measurements.

### X-Ray Photoelectron Spectroscopy

Before measurements, millimeter-sized glass samples were molded in epoxy and polished using SiC papers and anhydrous diamond suspensions (down to a particle size of 3 μm). The X-ray photoelectron spectroscopy measurements were then performed using a Hiden MAXIM SIMS system equipped with a Specs XR50 X-ray source and a Specs Phoibos 150 electron analyzer. The X-ray beam (Al anode) has a wavelength of λ = 0.83401 nm while the X-ray optics ensures signal collecting from a spot of ∼2 mm in diameter (i.e., from both epoxy and glass sample). For this reason, we restrict the XPS analysis to Zn, which is present only in the ZIF sample. Subsequent raw data analyses were performed using CasaXPS software by performing the standard energy corrections according to the expected C 1s transition (284.8 eV) and following background removal.

### Estimation of Liquid Fragility (*m*)

The liquid fragility was estimated based on the DSC data and a correlation between heating rate and fictive temperature (*T*_f_), which provides the activation energy for viscous flow (*E*_g_),^34^
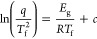
2

We determined *T*_f_ for heating rates of 10, 20, and 30 K min^–1^ for the sample prepared under a maximum pressure of 111.1 bar, and the activation energy was then determined from a linear fit to a plot of  against *T*_f_^–1^. The liquid fragility was finally determined as
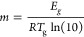
3

### *Ab Initio* Molecular Dynamics Simulations

To access the structural features of the liquid ZIF-water state, we performed *ab initio* molecular dynamics (MD) simulations of a unit cell of ZIF-62 with three added water molecules. This corresponds to the amount of water molecules in the ZIF volume when considering the volume of the experimentally used amount of water for the highest water content and the 100 μL crucible. In detail, the simulations were performed with the Vienna Ab initio Simulation Package (VASP) using the standard PBE pseudopotential and a time step of 0.5 fs. An energy cutoff of 400 eV and a convergence criterion of 10^–4^ eV were used. First, the water molecules were placed randomly in the ZIF unit cell using Packmol, and then the structure was relaxed while allowing relaxation of both unit cell dimensions and positions using a target pressure of 0. Next, dynamics were initiated at 1000 K and run for 10 ps (a total of 20 000 molecular dynamics steps). In this process, the structure was allowed to deform freely with a target pressure of 100 bar (to mimic the pressure inside the DSC crucible). The structure, especially of the bonding environments (neighbors and possible bonds) of the added water molecules, was evaluated throughout the simulation.

## Results and Discussion

### Melting Point Suppression

**Figure 1:**
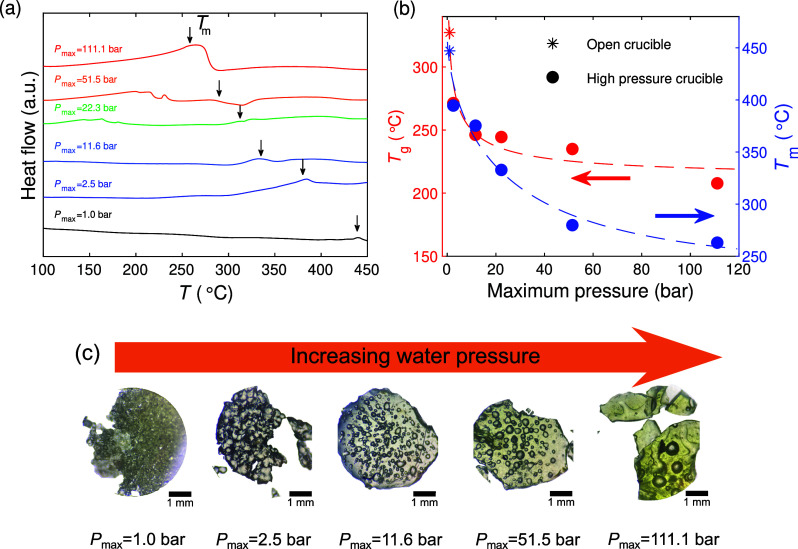
(a) Calorimetric heating scans of ZIF-62 crystals mixed with varying amounts of water, corresponding to maximum water pressures of 1.0, 2.5, 11.6, 22.3, 51.5, and 111.1 bar. (b) Melting temperature (*T*_m_) and glass transition temperature (*T*_g_) as a function of the maximum hydrothermal pressure. Open crucibles are regular aluminum DSC (max internal pressure of 3 bar) pans, while high-pressure crucibles are capable of withstanding internal pressures up to 270 bar. Arrows indicate relevant *y*-axis. (c) Optical micrographs of the formed glasses, showing an increase of fluidity with increasing water pressure. The scalebar in each micrograph corresponds to 1 mm.

In detail, we have mixed solvent-free crystalline ZIF-62 (see X-ray diffraction [XRD] pattern in Figure S6) with varying amounts of water, then sealed the crucible, and subsequently heated it to 460 °C without escape of water from the crucible. This creates large vapor pressures (up to ∼111 bar, see Methods and Figures S3 and S7) that in turn causes a giant reduction in *T*_m_ of ZIF-62 from ∼447 °C to below 300 °C (Figure 1a,b) and in *T*_g_ from ∼320 °C to ∼200 °C (Figures 1b and S8).

All the obtained glasses following melt-quenching under pressure are found to be noncrystalline and show no signs of decomposition based on XRD and ^1^H NMR analyses of the digested samples (Figures S9–11). All studied glasses have been subjected to similar heat treatment (linear increase to 460 °C followed by linear decrease to RT, all at 10 K min^–1^), but the high temperatures caused water evaporation *inside* the crucible (see Figures 1a and S7), creating hydrothermal pressures depending on the water content. While our crucibles are rated to ∼270 bar of pressure, we estimate the maximum experienced pressure, using the Antoine and ideal gas equations (see Figure S3), inside the crucible with the highest amount of water to be around *p* = 111.1 bar (∼10 MPa). This high hydrothermal pressure causes weaker calorimetric melting peaks (Figure 1a) as well as significant endothermal evaporation signals (Figures 1a and S7), making it difficult to accurately determine the *T*_m_ for the highest water contents. To determine *T*_m_ more accurately, we have heated two crystal-water samples to temperatures below and above the suspected transition temperatures to investigate if glass formation has occurred. Based on the recorded XRD patterns and sample morphology after the calorimetric measurements (Figures S12–13), we find that the sample with the highest water content (and pressure) undergoes complete vitrification already upon heating to 295 °C, confirming the assignment in Figure 1a. The sample with a lower water content also shows good agreement between the *T*_m_ value determined from calorimetry (Figure 1a) and the observed temperature of vitrification.

In addition to the presented calorimetry scans (Figures 1a and S8a of melting and glass transition behavior, respectively), we have also made the following two tests: 1) glass prepared under atmospheric pressure (i.e., in an Al crucible without water, shown as *P*_max_ = 1 bar in Figure 1a) is subsequently scanned in a closed high pressure crucible with water (amount corresponding to *P*_max_ ≈ 55 bar); and 2) glass prepared with the highest used water content (*P*_max_ = 111.1 bar in Figure 1a) is subsequently scanned in an Al crucible (*P*_max_ = 1 bar). This was done to access the impact of permanent and dynamic effects on the recorded glass transitions. Figure S14a shows that the glass prepared at ambient pressure features a significant drop in *T*_g_ to ∼232 °C upon rescanning with water (*P*_max_ ≈ 55 bar). This is similar to what is expected for a glass initially prepared with water (Figure 1b). In contrast, Figure S14b shows that rescanning a glass originally prepared with a high water vapor pressure (*P*_max_ = 111.1 bar) in an Al crucible reveals a glass transition temperature of ∼277 °C (compared to ∼200 °C when performing a scan with water), followed by a weight loss (∼1 wt %, see Figure S14c), which is likely due to water release as this has previously been found to be released around this temperature in the ZIF-62 crystal.^35^ These measurements suggest a permanent densification effect inducing a *T*_g_ drop of ∼45 °C (compared to *T*_g_ ∼ 320 °C when making the ZIF-62 glass without water), while the presence of water vapor induces another *T*_g_ drop of ∼70 °C. Notably, upon a subsequent upscan (after first scanning to 450 °C), the densification effect is relaxed, and we measure a *T*_g_ of ∼320 °C (Figure S14b). The change of *T*_g_ of the permanently densified glass is similar to that observed (Δ*T*_g_ ≈ 40 K) in a recent study of spark-plasma-sintered ZIF-62 glass compressed to 60 MPa (600 bar).^21^

**Figure 2:**
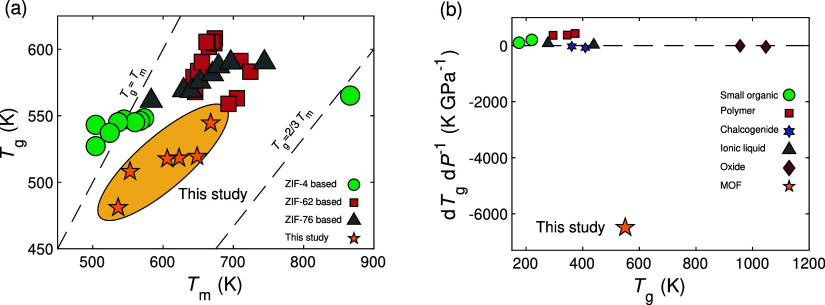
(a) Glass transition temperature (*T*_g_) as a function of melting temperature (*T*_m_) for various derivatives of ZIF-4 (green circles),^19^ ZIF-62 (red squares),^36,44^ and ZIF-76 (gray triangles)^36,45^ glasses as well as the present ZIF-62 glasses subjected to hydrothermal treatment (orange stars). (b) *In situ* pressure sensitivity of the glass transition temperature (d*T*_g_/d*P*) as a function of the glass transition temperature for a range of organic,^37−40^ chalcogenide,^41,42^ and inorganic glasses,^43^ as well as the studied ZIF-62 system for the highest pressure case.

Interestingly, optical microscopy images of the samples after initial melt-quenching with and without the presence of water reveal clear evidence of viscous flow, with the hydrothermal treatment giving rise to liquids of significantly increased fluidity (Figure 1c). This is explained by a significant drop in *T*_g_ (defined as the 10^12^ Pa·s isokom temperature) from the standard *T*_g_ value of ZIF-62 glass of ∼325 to ∼200 °C for the samples subjected to the largest hydrothermal pressure (Figure 1b). To our knowledge, the obtained values of *T*_m_ and *T*_g_ are the lowest ever reported for any melt-quenched ZIF system.^3,11,16,21,22,36^ We summarized *T*_g_ and *T*_m_ values for various ZIFs in Figure 2a. Similarly, we have estimated the effect of pressure on *T*_g_ and found a pressure sensitivity of d*T*_g_/d*P* ≈ −6500 K GPa^–1^ (Figure 2b). This value is based on the highest pressure value case while taking into account the permanent densification effect as identified in Figure S14. This pressure effect is notably larger than any studies of organic,^37−40^ chalcogenide,^41,42^ and inorganic glass (see examples in Figure 2b).^43^ We have also probed the liquid fragility index *m* (i.e., the rate of change of viscosity at *T*_g_) of the dried ZIF-62 system prepared at *P*_max_ = 111.1 bar (see Methods and Figure S15). From this, we find it to be a superstrong system with a liquid fragility of *m ≈* 18, even lower than that of the same glass prepared under ambient pressure (*m* ≈ 23).^22^

In addition to the performed analyses on the solvent-free ZIF-62 crystals, we have also investigated the effect of hydrothermal treatment on crystalline ZIF-62 with DMF solvent trapped in the pores after the synthesis (Figures S16–18). This analysis shows a significant drop in *T*_m_ upon hydrothermal treatment, but not to the same extent as the case of dried ZIF-62 (minimum *T*_m_ value of ∼350 °C for the undried case). Furthermore, no clear glass transition peak could be identified for these samples, but we did observe increased fluidity without decomposition upon hydrothermal treatment (Figure S17).

### Structure at Different Length Scales

In the following, we investigate whether the observed changes in *T*_m_ and *T*_g_ are due to the water-generated pressure inside the sealed crucible, structural incorporation of water in the ZIF, or a combination of these two effects. First, we note that a proposed ZIF-62 phase diagram suggests a reduction in *T*_m_ down to ∼360 °C at around 2 GPa of pressure,^20^ while another study found a reduction in *T*_g_ by 40 °C upon spark-plasma sintering at 0.06 GPa.^21^ Here, we have subjected crystalline ZIF-62 to significantly lower pressures than those in the previous studies (max pressure of ∼0.01 GPa), but nonetheless, we observe a significantly greater reduction in *T*_m_ and *T*_g_. This points to the importance of having water vapor compared to nonpenetrable mechanical pressure from, e.g., the spark plasma sintering press or the large molecular weight pressure medium from a diamond anvil cell.^20,21^ Furthermore, recent results on a water-sensitive bis-acetamide hybrid glass have demonstrated that water may be directly incorporated in the glass structure.^12^ Such water incorporation is also a possibility in the present ZIF-62 glass, but the calorimetry scans reveal an endothermic peak around 0 °C (Figure S8a), suggesting that free water (i.e., unbound H_2_O) is present in the system *after* glass formation and that the added water is thus not fully incorporated into the resulting glass network. This is further supported by the infrared spectroscopy analysis (Figure S19), revealing no major absorption in the characteristic region of water (or hydroxyl) vibrational modes (3000–4000 cm^–1^). We also note that the FTIR results suggest that there is no significant decomposition upon melt-quenching of the ZIF samples. X-ray photoelectron spectroscopy measurements also show no clear change in the Zn or N environments (Figures S20 and S21). Practically, we found a liquid (most likely water) to be condensing on the inside of the high-pressure crucible lid after the glass formation procedure was performed, separately from the produced glass, which would stick to the bottom of the crucible walls. This explains the difference between the amount of water added in the highest pressure cases (∼25 wt % of the total system mass) compared to the mass of the released water upon rescanning a sample prepared under high pressure in a low pressure crucible (∼1 wt %, see Figure S14c).

**Figure 3:**
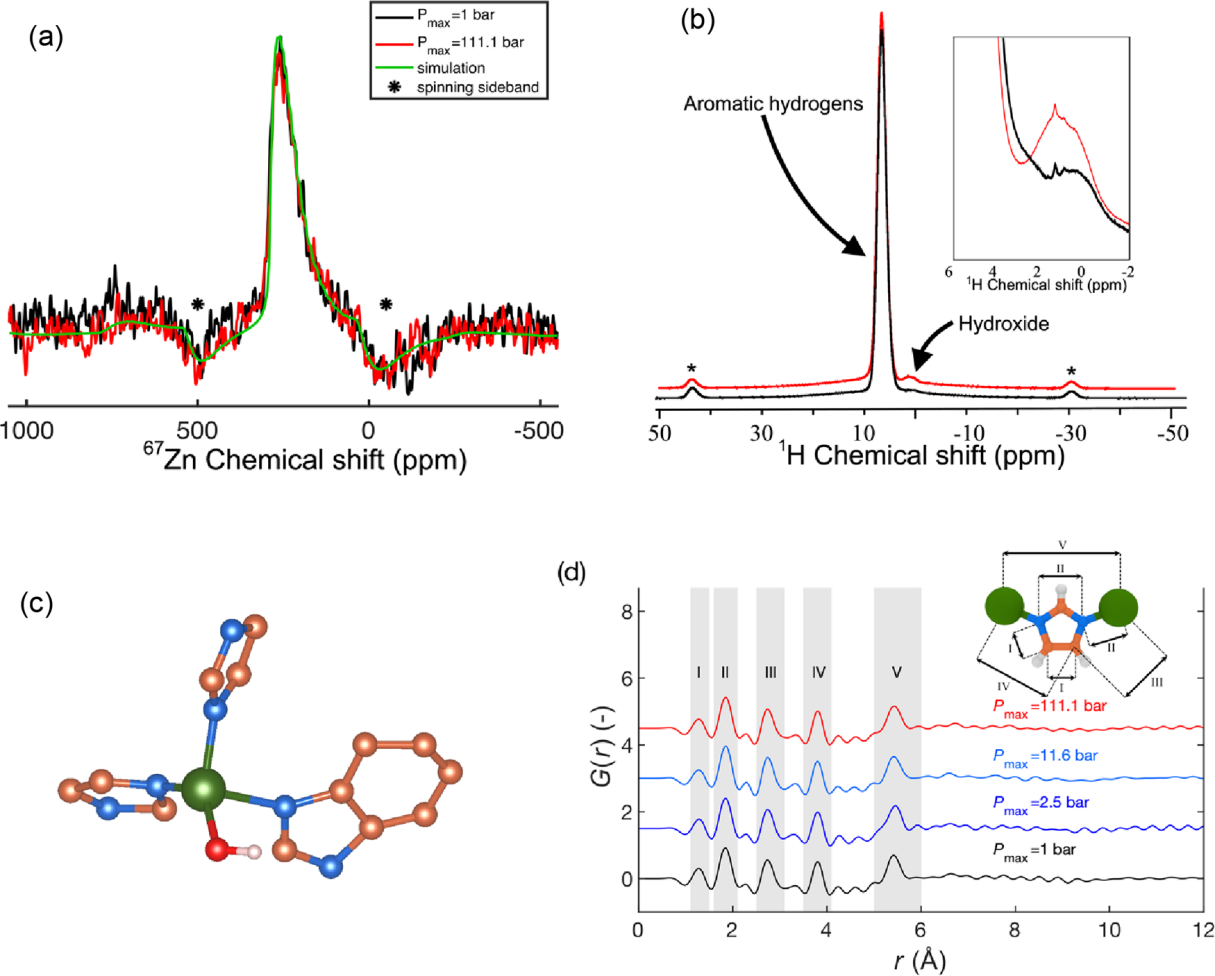
(a) Solid-state ^67^Zn MAS NMR spectra of samples prepared at 1 and 111.1 bar of hydrothermal pressure along with a numerical simulation of the spectra. (b) Solid-state ^1^H MAS NMR spectra of samples prepared at 1 and 111.1 bar of hydrothermal pressure. Inset shows the −2 to 6 ppm range, highlighting the signal from hydroxide (OH^–^). The data in the main plot are shifted vertically for the sake of clarity. Coloring is similar to that in panel (a). The stars (*) in both panels (a) and (b) indicate spinning sidebands. (c) *Ab initio* MD simulated atomic snapshot of configuration of hydroxide coordinating to a Zn polyhedra together with both imidazolate and benzimidazolate. Zn is green, N blue, C orange, O red, and H white. All aromatic hydrogens are omitted for clarity. (d) Pair distribution function, *G*(*r*), of ZIF-62 glasses prepared under argon and water gas pressures ranging from 1 to 111.1 bar. An illustration of the internal distances in the zinc-imidazolate-zinc ZIF building block is shown as an inset. Distances are labeled in roman numerals and correlated with the shaded peaks in the *G*(*r*) data.

To probe the network-forming short-range order in the formed glass, we target the local Zn-environment by ^67^Zn solid-state MAS NMR spectra recorded for ZIF-62 glass samples prepared at ambient pressure and at 111.1 bar under hydrothermal conditions (Figure 3a). This technique has previously been found to be very sensitive to short-range distortions in the Zn-tetrahedra.^33^ In Figure 3a we find, within the limitation of available signal-to-noise, overlapping signals of the two studied samples, which (as ZIF-62 glass in ref (33)) match very well with numerical SIMPSON^28^ simulations of the line-shape under consideration of Czjzek model distribution of quadrupolar coupling interactions as representative for disordered solids^32^ (see details on experiments and data treatment in Figures S4 and S5). This implies no major alteration of the local Zn-environment in either the sample prepared at ambient pressure or the sample prepared at hydrothermal pressures of up to 111.1 bar. We note that we find a stronger signal from the *P*_max_ = 111.1 bar sample. We ascribe this to a higher density of this sample (estimated Δρ ≈ 10% based on the packed amount of powder in the NMR rotors) as well as slight differences in the total measuring times. The signals in Figure 3a are normalized against sample scans and the mass.

We supplement the ^67^Zn NMR spectroscopy measurements with solid-state ^1^H MAS NMR measurements of the samples prepared at *P*_max_ values of 1 and 111.1 bar, respectively (Figure 3b). Here, we identify two main peaks, namely, a very large one arising from aromatic hydrogens from the imidazolate linkers (shift of 4–8 ppm) and a less intense one at around 1.5 ppm, which is especially apparent in the sample prepared at *P*_max_ = 111.1 bar of water pressure. Notably, the shift of this peak matches that expected for hydroxide ions (OH^–^).^46^ This suggests that the added water acts as an acid to donate a H^+^ to a bridging imidazolate/benzimidazolate, presumably leading to a (benz)imidazole only coordinating to one Zn polyhedra with OH^–^ acting as a new ligand. To further assess this hypothesis, we consider the *ab initio* MD simulation of a ZIF-62 unit cell loaded with three water molecules. We chose this ZIF-to-water ratio as it corresponds to the amount of water molecules in the volume occupied by the ZIF sample in the experiments if the water is evenly distributed in the 100 μL crucible. Upon relaxation and 10 ps of dynamics at 1000 K (see Methods), we find that one of the three water molecules has indeed lost one hydrogen atom and forms a stable coordination to a Zn polyhedron (Figure 3c). This supports the suggested mechanism. However, we note that only a minor fraction of the added water undergoes this reaction, since we find that the majority of the added water does not enter the structure (as observed from the similar calorimetry signals of water with and without ZIF in the closed crucible; see Figures S7 and S8a). The mechanism involving defective ZIF structures upon reaction with water is also supported by previous work on especially ZIF-8.^47−49^

To further study the structural impact of the hydrothermal treatment, we have measured total X-ray scattering and, therefrom, obtained the pair distribution functions (PDFs) of the ZIF-62 glasses prepared at ambient and elevated pressures (up to 111.1 bar). The PDFs are presented in Figure 3d (reciprocal space functions are shown in Figure S22). While the atom correlations I–IV (see Figure 3d) consist of several overlapping peaks from the (benz)imidazolate linkers, correlation V is clearly ascribed to the Zn–Zn correlation. Remarkably, this correlation shows little change with pressure (Figure 3d), suggesting that the overall structural effect of the hydrothermal pressure is relatively small (although it has a large effect on the transition temperatures). Considering the peak at the smallest *Q*-value in the X-ray structure factor *S*(*Q*), we observe a small shift in position toward higher *Q* with pressure (Figure S22b), which we ascribe to changing medium-range order packing.^21,50,51^ In turn, this is likely caused by the observed water vapor-assisted densification of the overall structure (as *Q* is inversely proportional to real space length).^33^ The lack of any obvious changes in the PDF caused by OH^–^ coordination can likely be ascribed to the following two effects. First, only a small fraction of (benz)imidazolate linkers appear to be exchanged. Second, the coordination of OH^–^ to Zn^2+^ is expected to be similar to that of the short-range correlations for Zn-imidazolate due to the very similar X-ray scattering lengths (i.e., the number of electrons) of oxygen and nitrogen.

To supplement the experiments using water, we have also tested the use of two other solvents as pressure media, specifically, ethanol (EtOH), and *n*-hexane. EtOH was chosen because it, like water, features lone pairs available for coordination bonding, whereas *n*-hexane was chosen because it is a noncoordinating molecule due to the lack of lone pairs. Notably, we find no major effect on the melting temperature upon EtOH or *n*-hexane addition for these samples (see Figure S23a), but we observe an apparent decrease of the melting enthalpy for the sample with *n*-hexane. We also observe peaks that could be interpreted as glass transitions in the range of 270–280 °C (see Figure S23b), indicative of glass formation caused by the pressure effect from the gaseous EtOH/*n*-hexane. However, unlike that with water, no giant suppression of *T*_m_ and *T*_g_ is found. We speculate that this may be due to the differences in kinetic diameters (water: ∼2.7 Å; ethanol and *n*-hexane: 4.4–4.5 Å),^52,53^ enabling water to enter the ZIF-62 structure (pore size ∼2.7–3.3 Å)^7^ while excluding EtOH and *n*-hexane. Lastly, we note that the visual appearance of the samples also differs significantly (Figure S24).

### Mechanical Properties

**Figure 4:**
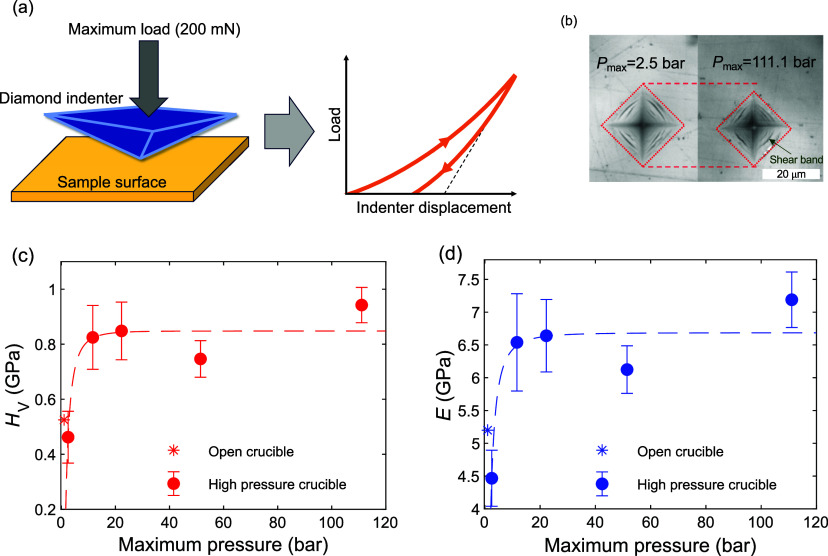
(a) Sketch of an indentation experiment. (b) Examples of residual indentation imprints on the surfaces of the samples experiencing a maximum pressure of 2.5 bar (left) and 111.1 bar (right) during the glass synthesis procedure (the indentation load was 200 mN). The white scalebar is 20 μm in length. (c,d) Indentation-based measurements of (c) Vickers hardness (*H*_V_) and (d) Young’s modulus (*E*) of the studied ZIF-62 glasses at water gas pressures ranging from 1 to 111.1 bar. The *H*_V_ and *E* data are also compared with previous literature reports, shown with star symbols in panels (c) and (d), acquired using microscopic analysis of indents and ultrasonic echography,^14,26^ respectively, on ZIF-62 synthesized in open crucibles (i.e., *P*_max_ = 1 bar, star symbol). Dashed lines in panels (b), (c), and (d) are guides for the eye.

Finally, we have performed mechanical characterization of the samples through depth-sensing indentation testing using a Vickers diamond tip (i.e., four-sided pyramid diamond indenter with a face angle of 136°). The resulting load-depth curves (sketched in Figure 4a) are used to determine Vicker’s hardness (*H*_V_) and Young’s modulus (*E*) of the samples using the Oliver–Pharr method.^24,25^ We provide a detailed description of the indentation procedure in the Methods section. Example of the residual imprints after unloading in the samples subjected to maximum water gas pressures of 2.5 and 111.1 bar during synthesis is shown in Figure 4b, revealing a significantly smaller imprint upon hydrothermal treatment, corresponding to an increase of hardness. Interestingly, we also find evidence of shear-banding in these samples (that is, zones of significant strain as exemplified with a green arrow in Figure 4b), similar to what has previously been observed for pristine ZIF-62 glass.^26^ The obtained values of *H*_V_ and *E* are shown in Figure 4c,d as extracted from load–displacement data (see Figure S25). The data reproduce previous reports^14,26^ in terms of the absolute values of *H*_V_ and *E* for the pristine ZIF-62 glass, but upon hydrothermal treatment, *H*_V_ and *E* increase by ∼100% and ∼50%, respectively, at a moderate maximum water vapor pressure (<20 bar).

We note that a slight increase in *H*_V_ (Δ*H*_V_ ≈ 0.1 GPa) and a decrease in *T*_g_ (Δ*T*_g_ ≈ −40 K) have previously been found for a spark-plasma sintered (SPS) ZIF-62 glass compressed to 600 bar.^21^ In comparison, the observed effect of hydrothermal pressure on *T*_g_, *H*_V_, and *E* in this work is more significant (Δ*T*_g_ of more than −100 K and Δ*H*_V_ > 0.4 GPa for the present study vs Δ*T*_g_ ≈ −40 K and Δ*H*_V_ < 0.1 GPa for the SPS study^21^) at pressures more than an order of magnitude lower (hydrothermal: <20 bar vs SPS: 600 bar). This stands in significant contrast to traditional glass families (e.g., oxides and chalcogenides), for which a decrease in *T*_g_ is typically associated with a decrease in network connectivity and thus a decrease in mechanical hardness and stiffness.^54−56^ As such, the present effect of decreasing *T*_g_ and increasing mechanical stiffness is fundamentally different from network modification effects in well-known glass formers.^21^ Here, we ascribe the property alterations to be caused by small exchange of organic linkers for hydroxide, the presence of guest molecules in the framework,^57^ changes in the medium-range order structures as hinted by the X-ray total scattering results (Figure S22) as well as an increase in density,^20,21^ but the question is now specifically how water governs the underlying densification mechanism compared to previous cases of densification in ZIF glasses.

### Densification Mechanism

While crystallographic analyses upon discovery of ZIF-62 suggested accessible pore sizes around 1.6 Å,^23^ it has later been found that CO_2_ (a kinetic diameter of ∼3.3 Å)^52^ can access both crystalline and glassy ZIF-62 structures.^58^ As the kinetic diameter of water (∼2.7 Å)^52^ is somewhat smaller than that of CO_2_, water should be able to physically enter the crystalline ZIF structure, effectively providing isostatic gaseous pressure inside the structure. In turn, this causes pore shape changes and “frustrates” the connected network, similar to the effect of adding bulky ligands.^17,19^ It is known that, e.g., the ZIF-8 structure is unstable in aqueous media due to cleavage of its Zn–N bond caused by the attack of water.^48^ We expect similar reactivity of other ZIF structures. Given the extreme environment inside the crucibles (up to 460 °C and >100 bar of water vapor pressure), the stability of the Zn–N bond is likely significantly impacted. While the water present in the crucible at lower temperatures is likely less reactive, at higher temperatures/pressures, the water vapor enhances the breakage of Zn–N bonds. Based on the results from solid-state ^1^H NMR and *ab initio* MD simulations, this is likely initiated by the creation of a new coordination bond between Zn and OH^–^ and possibly by water stabilizing the dangling (benz)imidazolate linkers. The presence of water in ZIF crystals thus causes more linker exchange at lower temperatures than that in the water-free ZIF crystals, thereby lowering the melting and glass transition temperatures of the crystalline and glassy structures, respectively. Furthermore, upon cooling, as evidenced by the solid-state ^1^H NMR data (Figure 3b) and MD simulations (Figure 3c), the hydroxide permanently exchanges a small amount of bridging (benz)imidazolate linkers in the framework.

This aligns well with how the introduction of various types of “disorder” (e.g., linker change, metal change, and internal strain from ball milling) has been found to lower the melting point in other MOFs.^17−19,59,60^ Interestingly, other available studies on pressure-related effects on ZIF-62 used either an isostatic high molecular weight (oil) pressure transmitting media^20^ or simple uniaxial mechanical pressing,^21^ while the present use of water is significantly different. This highlights the important role of the size and reactivity of the pressure-transmitting/generating medium, especially in the case of porous materials.

In addition, and opposite to observations in other glass-forming systems, hardness and elasticity are not positively correlated with *T*_g_, highlighting a decoupling of the glass dynamics and mechanics. While the introduction of hydroxide effectively breaks up the ZIF network, we suspect that the observed compression (Figure S22) and the presence of a guest molecule will induce more atomic “constraints”^61,62^ (i.e., fixation of atoms) per unit of volume—something which is well-known to cause an increase of both hardness and elasticity in the resulting glass phase.^54,56,63^ Simultaneously, the glass transition temperature is found to be permanently (drop in *T*_g_ of ∼40 K) and dynamically (further drop in *T*_g_ of ∼70 K) modified in the present system prepared with water. Considering the relation between *T*_g_ and hardness, we note that while hardness is a static property, the glass transition is associated with dynamical change of mobility in the underlying glass network as the temperature changes. As such, their decoupling is not necessarily surprising and may be associated with how the glass transition in the MOF glass systems tends to have a negative correlation with pressure—an effect which has previously been assigned to frustration and deformation of the pores inside the MOF structure.^21^ Furthermore, in the present case, the disturbance caused by the exchange of (benz)imidazolate for hydroxide may also disrupt the network dynamics (and thus *T*_g_) even at very small concentrations. A similar large effect on *T*_g_ upon only slight modifier addition is, e.g., found in alkali germanate glasses.^64^

## Conclusion

We have demonstrated that hot-compressed water significantly lowers the melting transition temperature of the zeolitic imidazolate framework ZIF-62, allowing glass formation at an unprecedented low temperature while simultaneously increasing hardness and mechanical stiffness by up to 100% of the resulting glass phase. We ascribe these effects to the water-assisted structural densification and reactivity of water inside the framework to partially replace bridging linkers for the hydroxide. The results shed light on the reactivity of water vapor with MOF systems at elevated temperatures and by doing so open a new avenue for the continuous alteration of both thermal and mechanical properties of MOF systems. We envision that the described water-assisted densification mechanism will enable the production of larger samples of more diverse hybrid chemistry as well as the tuning of properties of existing MOF families.

## Supporting Information Available

X-ray diffractograms of as-synthesized and dried ZIF-62 as well as all glasses, calorimetry scans of glasses and crucibles only containing water, liquid state ^1^H NMR of digested ZIF-62 crystal and glass samples, micrographs of glasses produced at varying temperatures, estimation of liquid fragility, X-ray total scattering, raw solid state NMR data and NMR modeling optimization, XPS and FTIR measurements of glasses, and finally load–displacement curves of glass samples from indentation experiments (PDF)

## References

[ref1] Horike S.; Shimomura S.; Kitagawa S. Soft Porous Crystals. Nat. Chem. 2009, 1 (9), 695–704. 10.1038/nchem.444. 21124356

[ref2] Bennett T. D.; Tan J. C.; Yue Y.; Baxter E.; Ducati C.; Terrill N. J.; Yeung H. H. M.; Zhou Z.; Chen W.; Henke S.; Cheetham A. K.; Greaves G. N. Hybrid Glasses from Strong and Fragile Metal-Organic Framework Liquids. Nat. Commun. 2015, 6, 8079 10.1038/ncomms9079. 26314784 PMC4560802

[ref3] Smedskjaer M. M.; So̷rensen S. S. A Glass Act. Nat. Chem. 2021, 13 (8), 723–724. 10.1038/s41557-021-00756-5. 34312505

[ref4] Banerjee R.; Phan A.; Wang B.; Knobler C.; Furukawa H.; O’Keeffe M.; Yaghi O. M.; Banerjee R.; Phan A.; Wang B.; Carolyn Knobler H. F.; O’Keeffe M.; Y O. M. High-Throughput Synthesis of Zeolitic Imidazolate Frameworks and Application to CO2 Capture. Science 2008, 319 (5865), 939–943. 10.1126/science.1152516. 18276887

[ref5] Bennett T. D.; Yue Y.; Li P.; Qiao A.; Tao H.; Greaves N. G.; Richards T.; Lampronti G. I.; Redfern S. A. T.; Blanc F.; Farha O. K.; Hupp J. T.; Cheetham A. K.; Keen D. A. Melt-Quenched Glasses of Metal-Organic Frameworks. J. Am. Chem. Soc. 2016, 138 (10), 3484–3492. 10.1021/jacs.5b13220. 26885940

[ref6] Yang Z.; Belmabkhout Y.; McHugh L. N.; Ao D.; Sun Y.; Li S.; Qiao Z.; Bennett T. D.; Guiver M. D.; Zhong C. ZIF-62 Glass Foam Self-Supported Membranes to Address CH4/N2 Separations. Nat. Mater. 2023, 22 (7), 888–894. 10.1038/s41563-023-01545-w. 37169976

[ref7] Wang Y.; Jin H.; Ma Q.; Mo K.; Mao H.; Feldhoff A.; Cao X.; Li Y.; Pan F.; Jiang Z. A MOF Glass Membrane for Gas Separation. Angew Chem., Int. Ed. 2020, 59 (11), 4365–4369. 10.1002/anie.201915807. 31893511

[ref8] Gao C.; Jiang Z.; Qi S.; Wang P.; Jensen L. R.; Johansen M.; Christensen C. K.; Zhang Y.; Ravnsbæk D. B.; Yue Y. Metal-Organic Framework Glass Anode with an Exceptional Cycling-Induced Capacity Enhancement for Lithium-Ion Batteries. Adv. Mater. 2022, 34, 2110048 10.1002/adma.202110048. 34969158

[ref9] Hou J.; Chen P.; Shukla A.; Krajnc A.; Wang T.; Li X.; Doasa R.; Tizei G.; H L.; Chan B.; Johnstone D. N.; Lin R.; Martens T. U.; Martens I.; Appadoo D.; Wang Z.; Wei T.; Lo S.-C.; Lu M.; Li S.; Namdas E. B.; Mali G.; Cheetham A. K.; Collins S. M.; Chen V.; Wang L.; Bennett T. D. Liquid-Phase Sintering of Lead Halide Perovskites and Metal-Organic Framework Glasses. Science 2021, 374, 621–625. 10.1126/science.abf4460. 34709926

[ref10] Ma N.; Horike S. Metal-Organic Network-Forming Glasses. Chem. Rev. 2022, 122 (3), 4163–4203. 10.1021/acs.chemrev.1c00826. 35044749

[ref11] Nozari V.; Calahoo C.; Tuffnell J. M.; Keen D. A.; Bennett T. D.; Wondraczek L. Ionic Liquid Facilitated Melting of the Metal-Organic Framework ZIF-8. Nat. Commun. 2021, 12, 5703 10.1038/s41467-021-25970-0. 34588462 PMC8481281

[ref12] So̷rensen S. S.; Ren X.; Du T.; Traverson A.; Xi S.; Jensen L. R.; Bauchy M.; Horike S.; Wang J.; Smedskjaer M. M. Water as a Modifier in a Hybrid Coordination Network Glass. Small 2023, 19 (14), 2205988 10.1002/smll.202205988. 36703506

[ref13] Ma N.; Horike N.; Lombardo L.; Kosasang S.; Kageyama K.; Thanaphatkosol C.; Kongpatpanich K.; Otake K. I.; Horike S. Eutectic CsHSO_4_-Coordination Polymer Glasses with Superprotonic Conductivity. J. Am. Chem. Soc. 2022, 144 (40), 18619–18628. 10.1021/jacs.2c08624. 36190375

[ref14] To T.; So̷rensen S. S.; Stepniewska M.; Qiao A.; Jensen L. R.; Bauchy M.; Yue Y.; Smedskjaer M. M. Fracture Toughness of a Metal-Organic Framework Glass. Nat. Commun. 2020, 11, 2593 10.1038/s41467-020-16382-7. 32444664 PMC7244719

[ref15] Varshneya A. K. Fundamentals of Inorganic Glasses; Elsevier, 2013.

[ref16] Nozari V.; Smirnova O.; Tuffnell J. M.; Knebel A.; Bennett T. D.; Wondraczek L. Low-Temperature Melting and Glass Formation of the Zeolitic Imidazolate Frameworks ZIF-62 and ZIF-76 Through Ionic Liquid Incorporation. Adv. Mater. Technol. 2022, 7 (11), 2200343 10.1002/admt.202200343.

[ref17] Frentzel-Beyme L.; Kloß M.; Kolodzeiski P.; Pallach R.; Henke S. Meltable Mixed-Linker Zeolitic Imidazolate Frameworks and Their Microporous Glasses: From Melting Point Engineering to Selective Hydrocarbon Sorption. J. Am. Chem. Soc. 2019, 141 (31), 12362–12371. 10.1021/jacs.9b05558. 31288513

[ref18] Madsen R. S. K.; Sarkar S.; Iversen B. B.; Yue Y. Sensitivity of the Glass Transition and Melting in a Metal–Organic Framework to Ligand Chemistry. Chem. Commun. 2022, 58 (6), 823–826. 10.1039/D1CC03541J. 34929725

[ref19] Song J.; Frentzel-Beyme L.; Pallach R.; Kolodzeiski P.; Koutsianos A.; Xue W. L.; Schmid R.; Henke S. Modulating Liquid-Liquid Transitions and Glass Formation in Zeolitic Imidazolate Frameworks by Decoration with Electron-Withdrawing Cyano Groups. J. Am. Chem. Soc. 2023, 145 (16), 9273–9284. 10.1021/jacs.3c01933. 37070213

[ref20] Widmer R. N.; Lampronti G. I.; Anzellini S.; Gaillac R.; Farsang S.; Zhou C.; Belenguer A. M.; Wilson C. W.; Palmer H.; Kleppe A. K.; Wharmby M. T.; Yu X.; Cohen S. M.; Telfer S. G.; Redfern S. A. T.; Coudert F. X.; MacLeod S. G.; Bennett T. D. Pressure Promoted Low-Temperature Melting of Metal–Organic Frameworks. Nat. Mater. 2019, 18 (4), 370–376. 10.1038/s41563-019-0317-4. 30886398

[ref21] Qiao A.; So̷rensen S. S.; Stepniewska M.; Biscio C. A. N.; Fajstrup L.; Wang Z.; Zhang X.; Calvez L.; Hung I.; Gan Z.; Smedskjaer M. M.; Yue Y. Hypersensitivity of the Glass Transition to Pressure History in a Metal – Organic Framework Glass. Chem. Mater 2022, 34 (11), 5030–5038. 10.1021/acs.chemmater.2c00325.

[ref22] Qiao A.; Bennett T. D.; Tao H.; Krajnc A.; Mali G.; Doherty C. M.; Thornton A. W.; Mauro J. C.; Greaves G. N.; Yue Y. A Metal-Organic Framework with Ultrahigh Glass-Forming Ability. Sci. Adv. 2018, 4 (3), eaao6827 10.1126/sciadv.aao6827. 29536040 PMC5844704

[ref23] Banerjee R.; Phan A.; Wang B.; Knobler C.; Furukawa H.; O’Keeffe M.; Yaghi O. M.; Banerjee R.; Phan A.; Wang B.; Carolyn Knobler H. F.; O’Keeffe M.; Y O. M.; O’Keeffe M.; Yaghi O. M. High-Throughput Synthesis of Zeolitic Imidazolate Frameworks and Application to CO_2_ Capture. Science 2008, 319 (5865), 939–943. 10.1126/science.1152516. 18276887

[ref24] Oliver W. C.; Pharr G. M. An Improved Technique for Determining Hardness and Elastic Modulus Using Load and Displacement Sensing Indentation Experiments. J. Mater. Res. 1992, 7 (6), 1564–1583. 10.1557/JMR.1992.1564.

[ref25] Franco Jr A. R.; Pintaúde G.; Sinatora A.; Pinedo C. E.; Tschiptschin A. P. The Use of a Vickers Indenter in Depth Sensing Indentation for Measuring Elastic Modulus and Vickers Hardness. Mater. Res. 2004, 7 (3), 483–491. 10.1590/S1516-14392004000300018.

[ref26] Stepniewska M.; Januchta K.; Zhou C.; Qiao A.; Smedskjaer M. M.; Yue Y. Observation of Indentation-Induced Shear Bands in a Metal-Organic Framework Glass. Proc. Natl. Acad. Sci. U. S. A. 2020, 117 (19), 10149–10154. 10.1073/pnas.2000916117. 32341165 PMC7229652

[ref27] Juhás P.; Davis T.; Farrow C. L.; Billinge S. J. L. PDFgetX3: A Rapid and Highly Automatable Program for Processing Powder Diffraction Data into Total Scattering Pair Distribution Functions. J. Appl. Crystallogr. 2013, 46 (2), 560–566. 10.1107/S0021889813005190.

[ref28] Bak M.; Rasmussen J. T.; Nielsen N. C. SIMPSON: A General Simulation Program for Solid-State NMR Spectroscopy. J. Magn. Reson. 2000, 147, 296–330. 10.1006/jmre.2000.2179. 11097821

[ref29] Zaremba S. K. Good Lattice Points, Discrepancy, and Numerical Integration. Ann. Mater. Pure. Appl. 1966, 4–73, 293 10.1007/BF02415091.

[ref30] Conroy H. Molecular Schrödinger Equation. VIII. A New Method for the Evaluation of Multidimensional Integrals. J. Chem. Phys. 2004, 47 (12), 5307–5318. 10.1063/1.1701795.

[ref31] Cheng V. B.; Suzukawa Jr H. H.; Wolfsberg M. Investigations of a Nonrandom Numerical Method for Multidimensional Integration. J. Chem. Phys. 2003, 59 (8), 3992–3999. 10.1063/1.1680590.

[ref32] d’Espinose de Lacaillerie J. B.; Fretigny C.; Massiot D. MAS NMR Spectra of Quadrupolar Nuclei in Disordered Solids: The Czjzek Model. J. Magn. Reson. 2008, 192 (2), 244–251. 10.1016/j.jmr.2008.03.001. 18362082

[ref33] Madsen R. S. K.; Qiao A.; Sen J.; Hung I.; Chen K.; Gan Z.; Sen S.; Yue Y. Ultrahigh-Field ^67^Zn NMR Reveals Short-Range Disorder in Zeolitic Imidazolate Framework Glasses. Science 2020, 367 (6485), 1473–1476. 10.1126/science.aaz0251. 32217725 PMC7325427

[ref34] Zheng Q.; Zhang Y.; Montazerian M.; Gulbiten O.; Mauro J. C.; Zanotto E. D.; Yue Y. Understanding Glass through Differential Scanning Calorimetry. Chem. Rev. 2019, 119 (13), 7848–7939. 10.1021/acs.chemrev.8b00510. 31120738

[ref35] Smirnova O.; Hwang S.; Sajzew R.; Ge L.; Reupert A.; Nozari V.; Savani S.; Chmelik C.; Reithofer M. R.; Wondraczek L.; Kärger J.; Knebel A. Precise Control over Gas-Transporting Channels in Zeolitic Imidazolate Framework Glasses. Nat. Mater. 2024, 23, 262 10.1038/s41563-023-01738-3. 38123813 PMC10837076

[ref36] Bumstead A. M.; Thorne M. F.; Bennett T. D. Identifying the Liquid and Glassy States of Coordination Polymers and Metal-Organic Frameworks. Faraday Discuss. 2021, 225 (Im), 210–225. 10.1039/D0FD00011F. 33104136

[ref37] Adrjanowicz K.; Kaminski K.; Koperwas K.; Paluch M. Negative Pressure Vitrification of the Isochorically Confined Liquid in Nanopores. Phys. Rev. Lett. 2015, 115, 265702 10.1103/PhysRevLett.115.265702. 26765007

[ref38] Bianchi U.; Turturro A.; Basile G. Pressure Effects on Glass Transition in Polymers Pressure Effects on Glass Transition in Polymers II.1 A Study of the Factors Affecting DTg/DP Values. https://pubs.acs.org/sharingguidelines.

[ref39] Koperwas K.; Grzybowski A.; Tripathy S. N.; Masiewicz E.; Paluch M. Thermodynamic Consequences of the Kinetic Nature of the Glass Transition. Sci. Rep. 2016, 5, 17782 10.1038/srep17782. PMC4674716 26657017

[ref40] Rzoska S. J. New Challenges for the Pressure Evolution of the Glass Temperature. Front. Mater. 2017, 4, 33 10.3389/fmats.2017.00033.

[ref41] Ramesh K. Pressure Dependence of Glass Transition in As2Te3 Glass. J. Phys. Chem B 2014, 118 (29), 8848–8853. 10.1021/jp504290z. 24962870

[ref42] Ramesh K.; Naresh N.; Pumlianmunga P.; Gopal E. S. R. Shift of Glass Transition Temperature under High Pressure for Ge20Te80 Glass. Key Eng. Mater. 2016, 702, 43–47. 10.4028/www.scientific.net/KEM.702.43.

[ref43] Bagdassarov N. S.; Naumus J.; Poe B.; Slutskiy A. B.; Bulatov V. K. Pressure Dependence of Tg in Silicate Glasses from Electrical Impedance Measurement. Phys. Chem. Glasses 2004, 45, 197–214.

[ref44] Thorne M. F.; Gómez M. L. R.; Bumstead A. M.; Li S.; Bennett T. D. Mechanochemical Synthesis of Mixed Metal, Mixed Linker, Glass-Forming Metal-Organic Frameworks. Green Chem. 2020, 22 (8), 2505–2512. 10.1039/D0GC00546K.

[ref45] Zhou C.; Longley L.; Krajnc A.; Smales G. J.; Qiao A.; Erucar I.; Doherty C. M.; Thornton A. W.; Hill A. J.; Ashling C. W.; Qazvini O. T.; Lee S. J.; Chater P. A.; Terrill N. J.; Smith A. J.; Yue Y.; Mali G.; Keen D. A.; Telfer S. G.; Bennett T. D. Metal-Organic Framework Glasses with Permanent Accessible Porosity. Nat. Commun. 2018, 9 (1), 1–9. 10.1038/s41467-018-07532-z. 30487589 PMC6262007

[ref46] Hou J.; Ashling C. W.; Collins S. M.; Krajnc A.; Zhou C.; Longley L.; Johnstone D. N.; Chater P. A.; Li S.; Coulet M.-V.; et al Metal-Organic Framework Crystal-Glass Composites. Nat. Commun. 2019, 10 (1), 2580 10.1038/s41467-019-10470-z. 31189892 PMC6561910

[ref47] Taheri M.; Tsuzuki T. Photo-Accelerated Hydrolysis of Metal Organic Framework ZIF-8. ACS Mater. Lett. 2021, 3 (2), 255–260. 10.1021/acsmaterialslett.0c00522.

[ref48] Zhang H.; Zhao M.; Yang Y.; Lin Y. S. Hydrolysis and Condensation of ZIF-8 in Water. Microporous. Mesoporous. Mater. 2019, 288, 109568 10.1016/j.micromeso.2019.109568.

[ref49] Zhang C.; Han C.; Sholl D. S.; Schmidt J. R. Computational Characterization of Defects in Metal-Organic Frameworks: Spontaneous and Water-Induced Point Defects in ZIF-8. J. Phys. Chem. Lett. 2016, 7 (3), 459–464. 10.1021/acs.jpclett.5b02683. 26771275

[ref50] Mei Q.; Benmore C. J.; Sen S.; Sharma R.; Yarger J. L. Intermediate Range Order in Vitreous Silica from a Partial Structure Factor Analysis. Phys. Rev. B 2008, 78, 144204 10.1103/PhysRevB.78.144204.

[ref51] So̷rensen S. S.; Biscio C. A. N.; Bauchy M.; Fajstrup L.; Smedskjaer M. M. Revealing Hidden Medium-Range Order in Amorphous Materials Using Topological Data Analysis. Sci. Adv. 2020, 6, eabc2320 10.1126/sciadv.abc2320. 32917687 PMC11206462

[ref52] Ismail A. F.; Khulbe K. C.; Takeshi M. Gas Separation Membranes Polymeric and Inorganic; Springer, 2015.

[ref53] Ferreira A. F. P.; Mittelmeijer-Hazeleger M. C.; Granato M. A.; Martins V. F. D.; Rodrigues A. E.; Rothenberg G. Sieving Di-Branched from Mono-Branched and Linear Alkanes Using ZIF-8: Experimental Proof and Theoretical Explanation. Phys. Chem. Chem. Phys. 2013, 15 (22), 8795–8804. 10.1039/c3cp44381g. 23640581

[ref54] Wilkinson C. J.; Zheng Q.; Huang L.; Mauro J. C. Topological Constraint Model for the Elasticity of Glass-Forming Systems. J. Non-Cryst. Solids: x 2019, 2, 100019 10.1016/j.nocx.2019.100019.

[ref55] Smedskjaer M. M.; Mauro J. C.; Yue Y. Prediction of Glass Hardness Using Temperature-Dependent Constraint Theory. Phys. Rev. Lett. 2010, 105, 115503 10.1103/PhysRevLett.105.115503. 20867584

[ref56] Yang K.; Yang B.; Xu X.; Hoover C.; Smedskjaer M. M.; Bauchy M. Prediction of the Young’s Modulus of Silicate Glasses by Topological Constraint Theory. J. Non. Cryst. Solids 2019, 514, 15–19. 10.1016/j.jnoncrysol.2019.03.033.

[ref57] Yang K.; Zhou G.; Xu Q. The Elasticity of MOFs under Mechanical Pressure. RSC Adv. 2016, 6, 37506–37514. 10.1039/C5RA23149C.

[ref58] Frentzel-Beyme L.; Kolodzeiski P.; Weiß J. B.; Schneemann A.; Henke S. Quantification of Gas-Accessible Microporosity in Metal-Organic Framework Glasses. Nat. Commun. 2022, 13, 7750 10.1038/s41467-022-35372-5. 36517486 PMC9751146

[ref59] Song J.; Pallach R.; Frentzel-Beyme L.; Kolodzeiski P.; Kieslich G.; Vervoorts P.; Hobday C. L.; Henke S. Tuning the High-Pressure Phase Behaviour of Highly Compressible Zeolitic Imidazolate Frameworks: From Discontinuous to Continuous Pore Closure by Linker Substitution. Angew Chem., Int. Ed. 2022, 61 (21), e202117565 10.1002/anie.202117565. PMC9401003 35119185

[ref60] Frentzel-Beyme L.; Kloß M.; Pallach R.; Salamon S.; Moldenhauer H.; Landers J.; Wende H.; Debus J.; Henke S. Porous Purple Glass-a Cobalt Imidazolate Glass with Accessible Porosity from a Meltable Cobalt Imidazolate Framework. J. Mater. Chem. A 2019, 7 (3), 985–990. 10.1039/C8TA08016J.

[ref61] Thorpe M. F. Continuous Deformations in Random Networks. J. Non. Cryst. Solids 1983, 57 (3), 355–370. 10.1016/0022-3093(83)90424-6.

[ref62] Mauro J. C. Topological Constraint Theory of Glass. Am. Ceram. Soc. Bull. 2011, 90 (4), 31–37.

[ref63] Zheng Q.; Yue Y.; Mauro J. C. Density of Topological Constraints as a Metric for Predicting Glass Hardness. Appl. Phys. Lett. 2017, 111, 011907 10.1063/1.4991971.

[ref64] Ashton-Patton M. M. Properties of Mixed Alkali Germanate Glasses. Ph.D.; Alfred University, 2013.

