# Supplementary material for: Water Promotes Melting of a Metal–Organic Framework
Source: Chem Mater. 2024 Mar 6;36(6):2756–66. doi: 10.1021/acs.chemmater.3c02873 (PMC10976635; doi:10.1021/acs.chemmater.3c02873)
Supplement: Supplementary file 1 — cm3c02873_si_001.pdf [file cm3c02873_si_001.pdf]

Supporting Information

for

Water promotes melting of a metal-organic framework

Søren S. Sørensen[1,], Anders K. R. Christensen[1,†], Elena A. Bouros-Bandrabur[1,†], Emil S. Andersen[1,†], Heidi F. Christiansen[1,†], Sofie Lang[1,†], Fengming Cao[1], M. Faizal Ussama Jalaludeen[1], Johan F. S. Christensen[1], Wessel M. W. Winters[1], Bettina P. Andersen[2], Anders B. Nielsen[2], Niels Chr. Nielsen[2,3], Dorthe B. Ravnsbæk[2], Peter K. Kristensen[4], Yuanzheng Yue[1], Morten M. Smedskjaer[1,*]*

[1]Department of Chemistry and Bioscience, Aalborg University, DK-9220 Aalborg, Denmark

[2]Department of Chemistry, Aarhus University, DK-8000 Aarhus, Denmark

[3]Interdisciplinary Nanoscience Center (iNANO), Aarhus University, DK-8000 Aarhus, Denmark

[4]Department of Materials and Production, Aalborg University, DK-9220 Aalborg, Denmark

† These authors contributed equally

* Corresponding Authors.

Emails: Søren S. Sørensen soe@bio.aau.dk; Morten M. Smedskjaer mos@bio.aau.dk

Figure S1

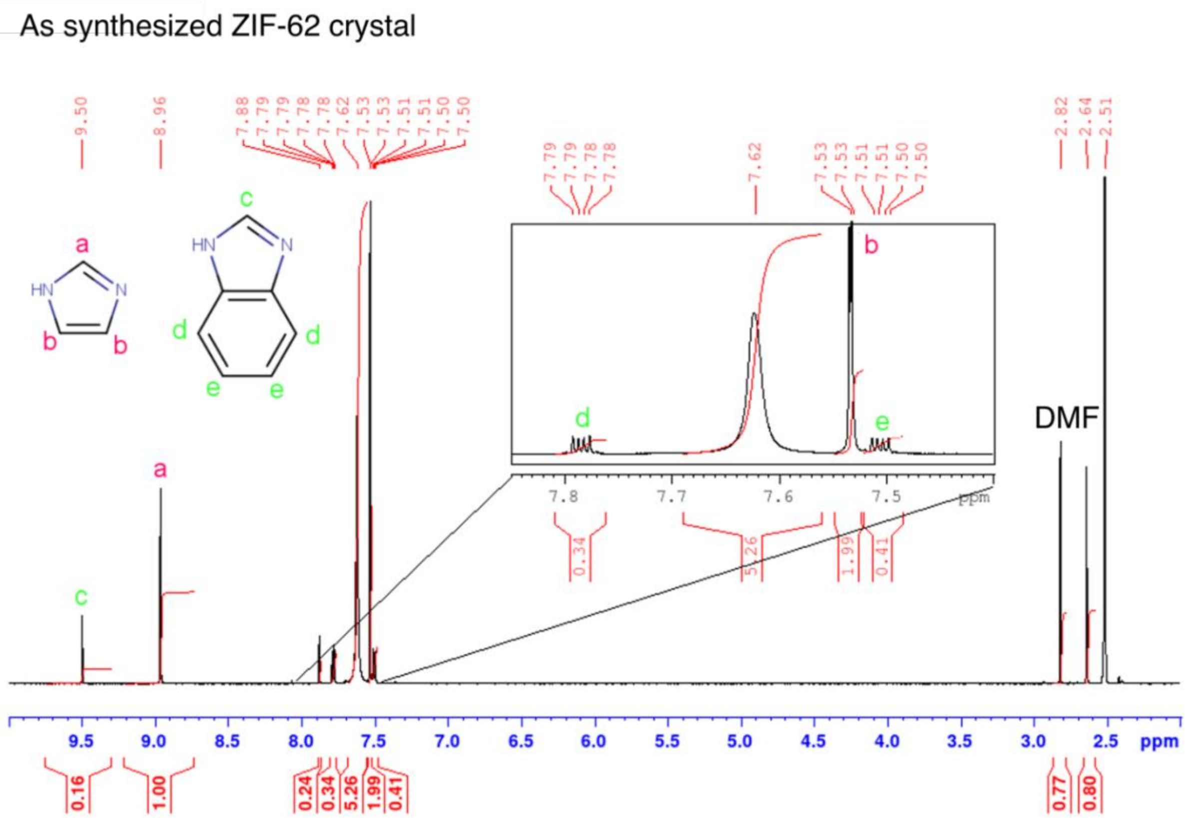

Figure S1. ^{1}H NMR spectrum of the as-synthesized ZIF-62 crystal digested in a mixture of DCl, D_2O, and DMSO-d_6 (see Methods for details). Peak assignments are given for protons in imidazolate (a, b) and benzimidazolate (c, d, e). Two peaks from the two chemically distinct methyl groups in dimethylformamide (DMF) are present in the 2.6-2.9 ppm region. The spectrum integration is normalized according to the singlet originating from the a-proton in imidazolate (~9 ppm)

Figure S2

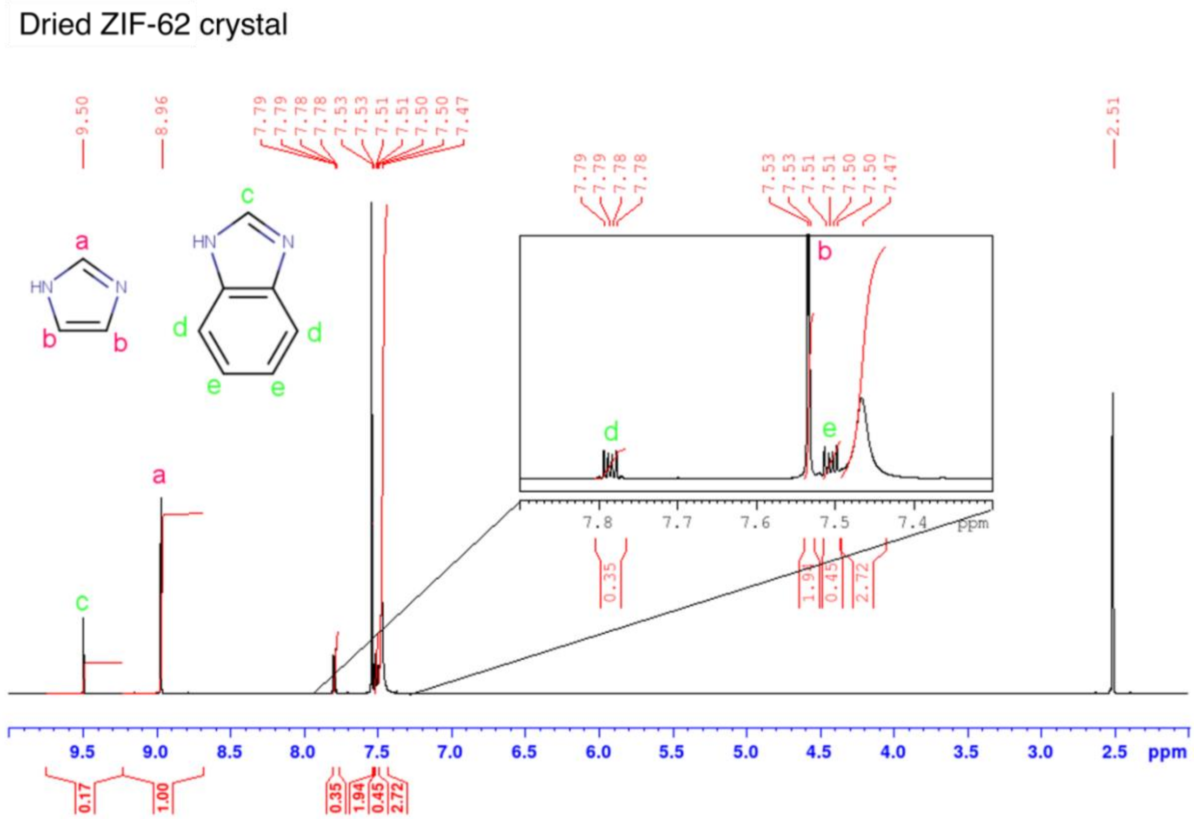

Figure S2. ^{1}H NMR spectrum of the dried ZIF-62 crystal digested in a mixture of DCl, D_2O, and DMSO-d_6 (see Methods for details). Peak assignments are given for protons in imidazolate (a, b) and benzimidazolate (c, d, e). The spectrum integration is normalized according to the singlet originating from the a-proton in imidazolate (~9 ppm).

Figure S3

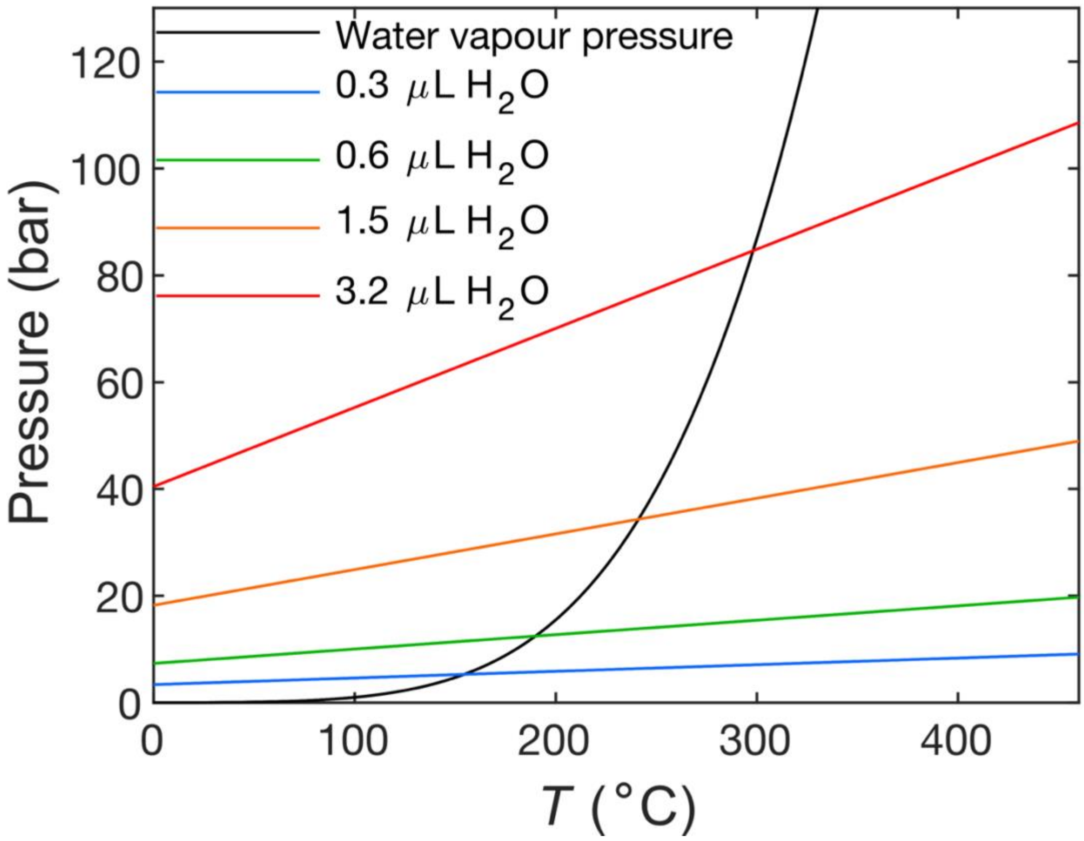

Figure S3. Estimation of the temperature dependence of the vapor pressure of water (H_2O) as determined using the Antoine equation (black line) as well as internal pressures in the 100 μL crucibles containing 0.3 (blue), 0.6 (green), 1.5 (orange), and 3.2 μL (red) of water, respectively, as calculated solely by the ideal gas equation ($P=nRT/V$). The pressure inside each water-containing crucible can be estimated as the lowest value of either the Antoine equation or the ideal gas law. Complete water evaporation is expected to occur at the temperature of intersection of the black and colored lines. This estimate agrees well with the temperatures of the endothermic peaks found in Figures 1a, S8, and S14a.

Figure S4

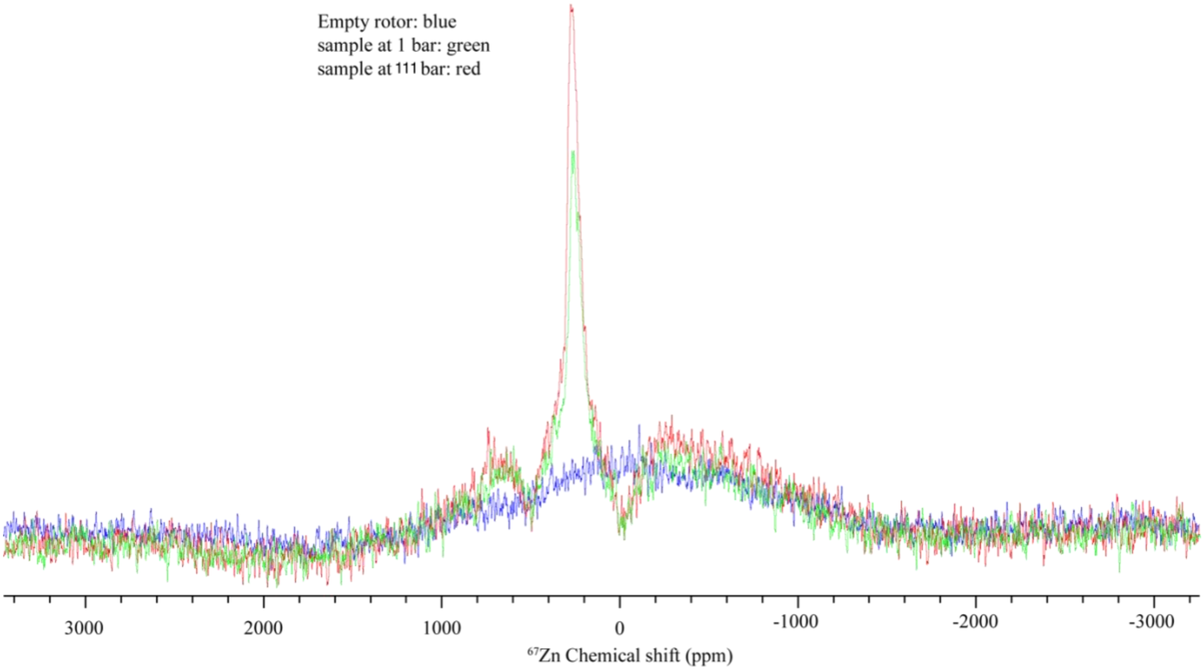

Figure S4. Raw data of ^{67}Zn solid state nuclear magnetic resonance measurements of samples prepared at 1 bar (green) and 111.1 bar (red) as well as the empty rotor (blue).

Figure S5

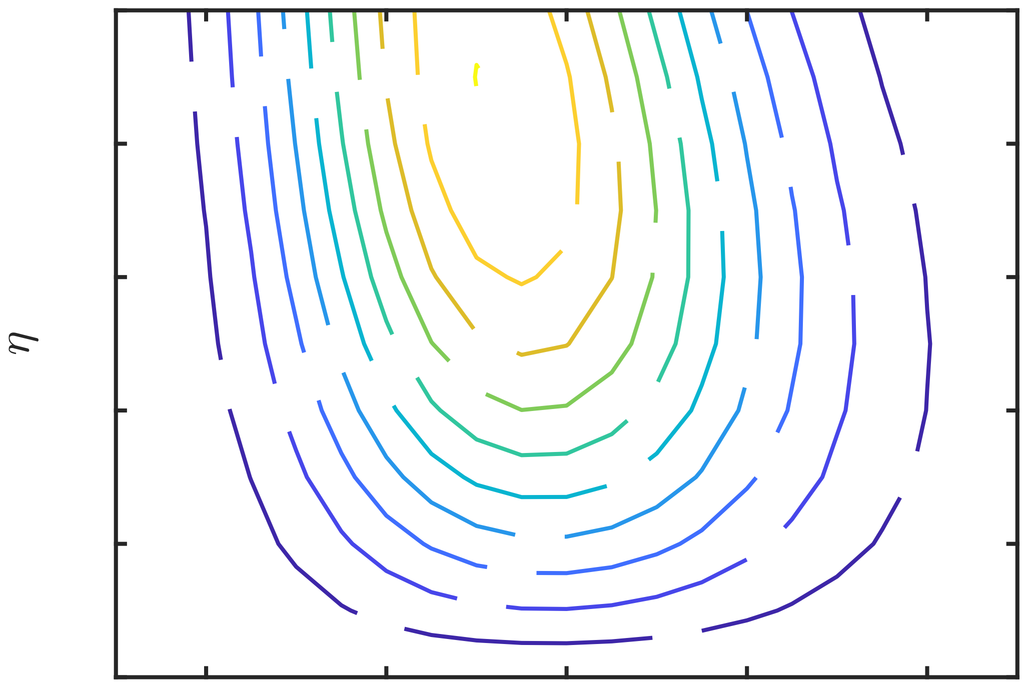

Figure S5. Czjzek model[1] weightning $P_Q(\nu_Q,\eta)$ (normalized; see formulas in Supporting Ref. 1) of a grid of SIMPSON simulated ^{67}Zn MAS NMR spectra of ZIF-62 samples (see Fig. 3a) calculated for a grid of C_Q and η parameters with $<C^2_{Q\eta}>$=6.5 MHz (see Methods section in the main text).

Figure S6

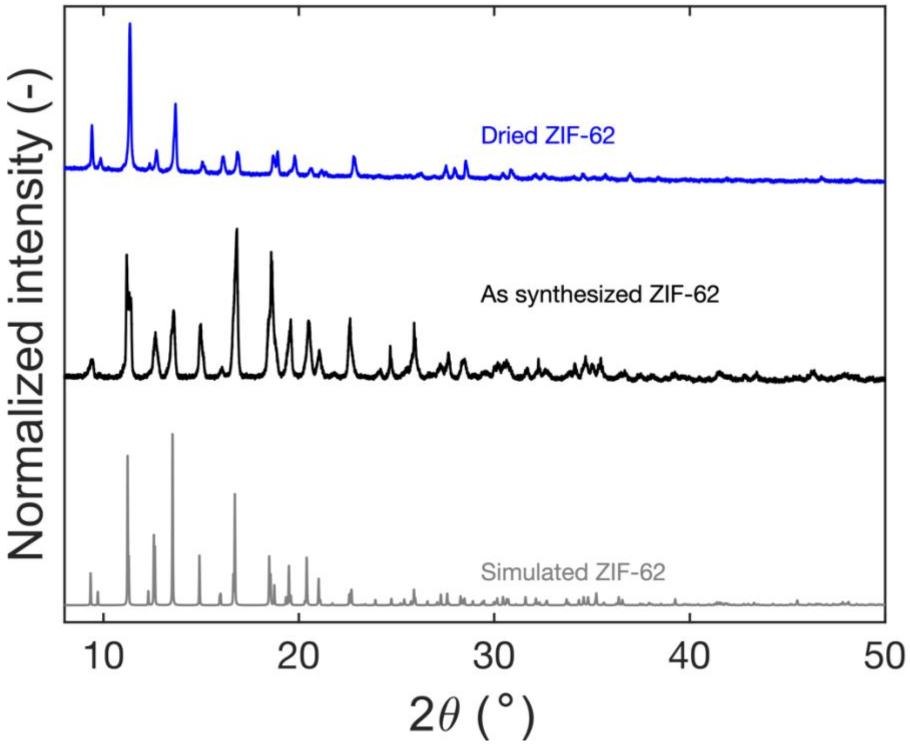

Figure S6. X-ray diffractograms of crystalline ZIF-62 as simulated from the published CIF file[2] (light grey, CCDC entry 671070) as well as from the as-synthesized ZIF-62 (black) and dried ZIF-62 (blue) crystals. Drying was performed at 350°C under an inert atmosphere.

Figure S7

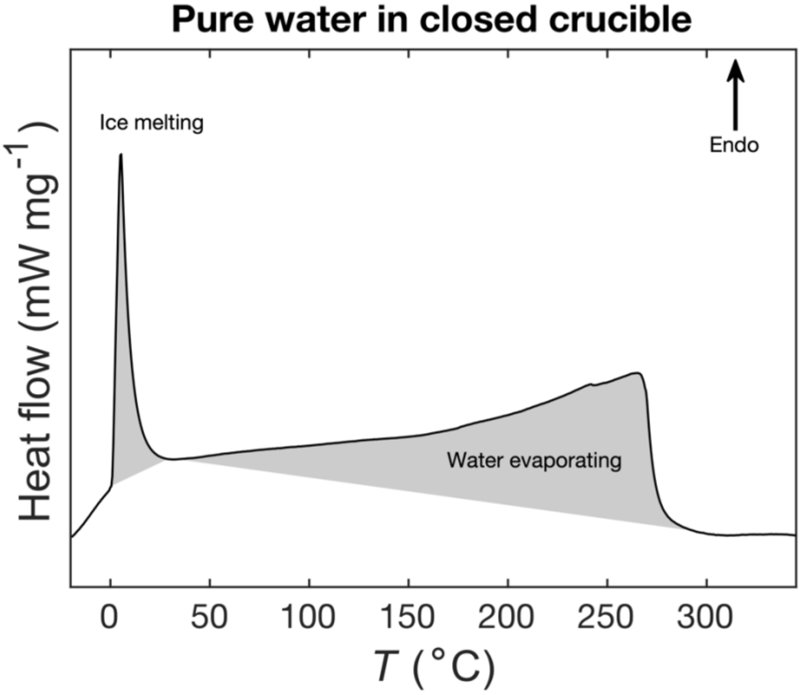

Figure S7. Differential scanning calorimetry (DSC) heating measurement (10 K min^{-1}) on ~3.2 µL H_2O sealed in a 100 µL high-pressure crucible to avoid evaporation. This amount of added water is equivalent to the water volume added to the samples featuring the highest maximum pressure of ~111.1 bar. Distinct peaks are clearly observed for the melting of ice around 0 °C and a broad endothermal response in the temperature region of 50-275°C related to the evaporation of the water inside the crucible. The mass of the crucible is stable during the experiment.

Figure S8

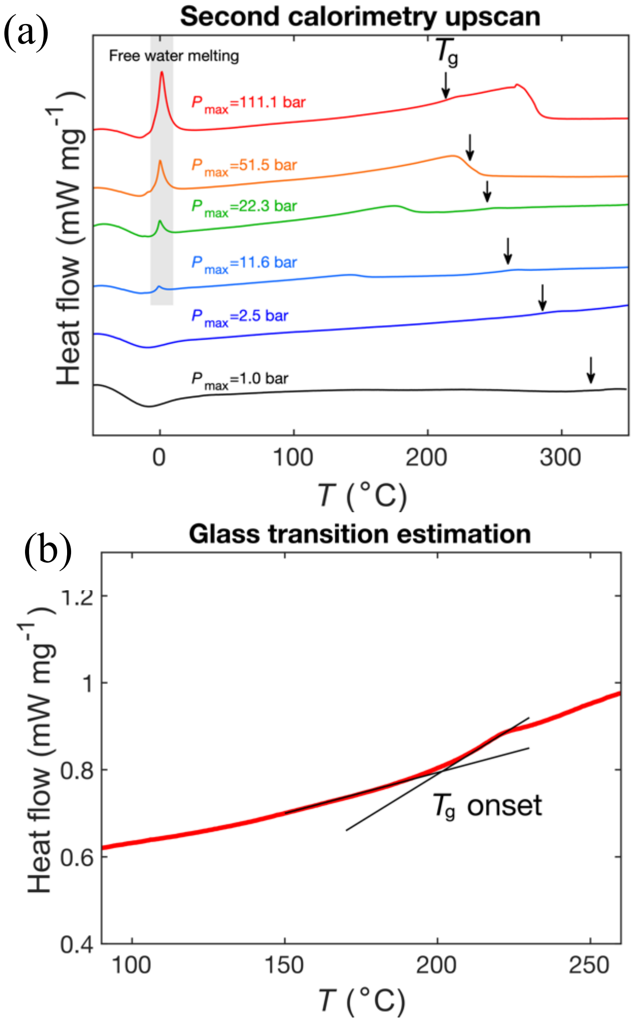

Figure S8. (a) Calorimetry heating scans of pre-formed glasses with different amounts of added water, corresponding to hydrothermal pressures up to 111.1 bar. All measurements were performed in the same high-pressure crucible as during glass formation without opening. A significant decrease in T_g with pressure is observed. For glasses with water remaining in the crucible, a clear endothermal peak is found around 0 °C, indicating the presence of chemically free water. A clear "nose" formed by the evaporation of water is also observed in the range of 150-300 °C, depending on the water content. (b) Example of estimation of glass transition temperature (T_g) of the sample with the highest water content (P_{max}=111.1 bar). The data is the same as presented in panel (a).

Figure S9

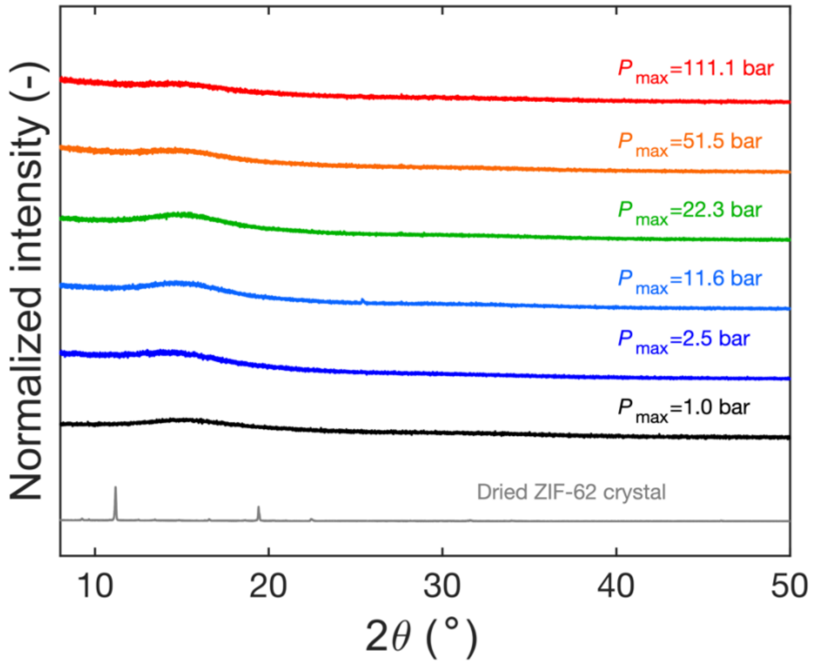

Figure S9. Normalized X-ray diffractograms of ZIF-62 glasses formed at various pressures (from 1 to 111.1 bar). All samples formed at ≥11.6 bar contained water as the pressure transmitting/generating medium. A spectrum of the dried ZIF-62 crystal (light grey) is also shown.

Figure S10

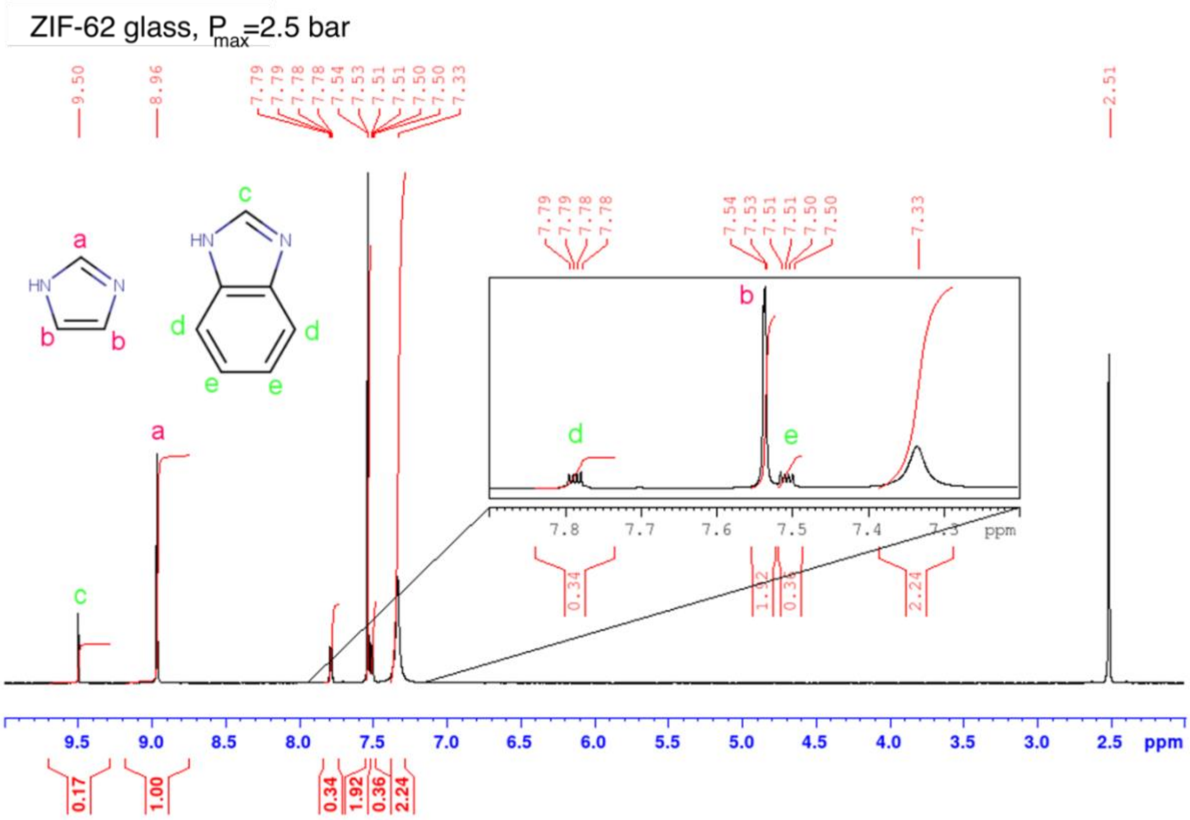

Figure S10. ^{1}H NMR spectrum of the dried ZIF-62 glass prepared at a maximum pressure of 2.5 bar (without water addition). The sample was digested in a mixture of DCl, D_2O, and DMSO-d_6 (see Methods for details). Peak assignments are given for protons in imidazolate (a, b) and benzimidazolate (c, d, e). The spectrum integration is normalized according to the singlet originating from the a-proton in imidazolate (~9 ppm)

Figure S11

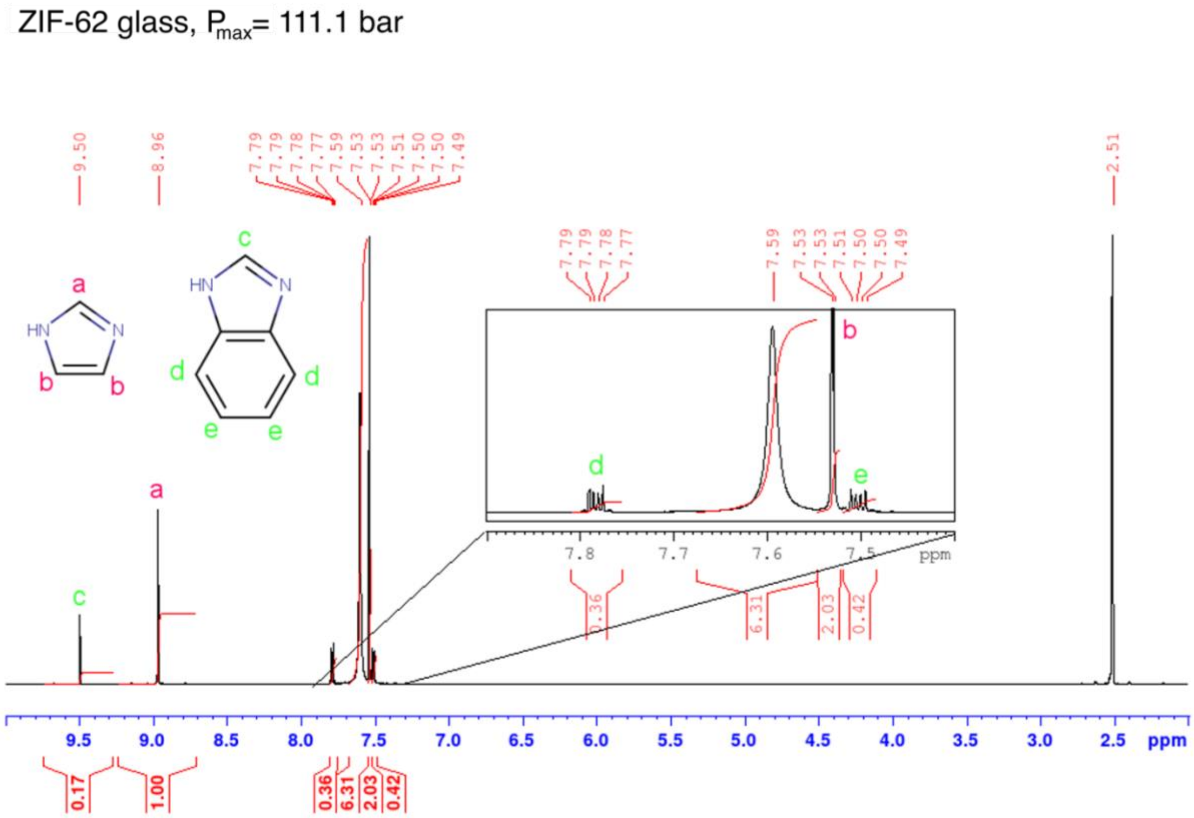

Figure S11. ^{1}H NMR spectrum of the dried ZIF-62 glass prepared at a maximum pressure of 111.1 bar (with water addition). The sample was digested in a mixture of DCl, D_2O, and DMSO-d_6 (see Methods for details). Peak assignments are given for protons in imidazolate (a, b) and benzimidazolate (c, d, e). The spectrum integration is normalized according to the singlet originating from the a-proton in imidazolate (~9 ppm).

Figure S12

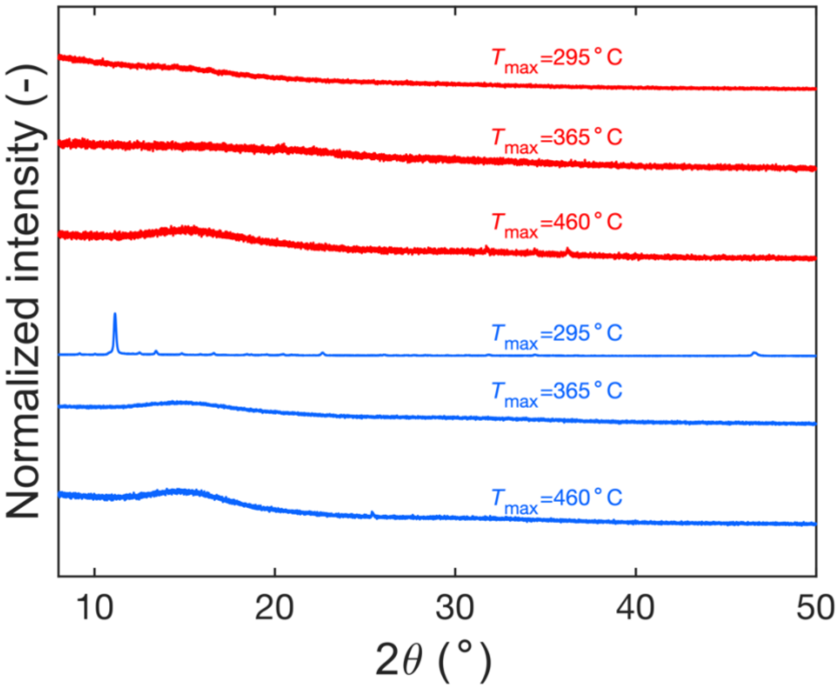

Figure S12. X-ray diffractograms of ZIF-water mixtures with similar water contents as samples reaching a maximum pressure of (blue) 11.6 and (red) 111.1 bar if heated to a temperature of T=460°C. The sample with a P_{max}=11.6 bar at T=460°C becomes non-crystalline between 295 and 365°C. Associated micrographs of samples are presented in Figure S13.

Figure S13

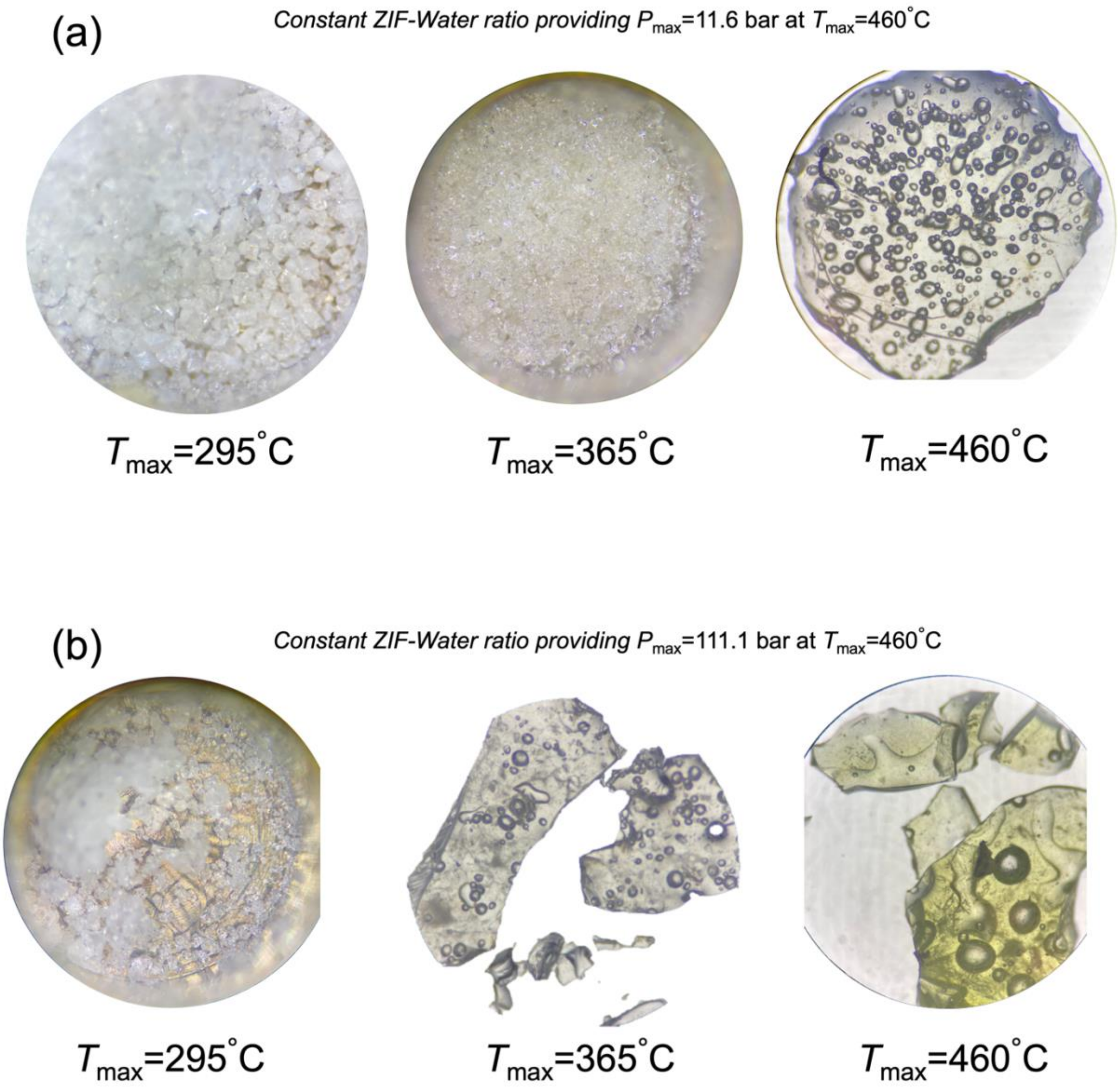

Figure S13. Micrographs of ZIF-water mixture samples with similar water contents as samples reaching (a) 11.6 and (b) 111.1 bar if heated to a temperature of T=460°C. In both panels (a) and (b), the sample experiencing the P_{max} is shown rightmost while two equivalent compositions are shown to the left and in the middle, only heated to ~295°C and ~365°C, respectively. In both (a) and (b) the field of view is ~5 mm.

Figure S14

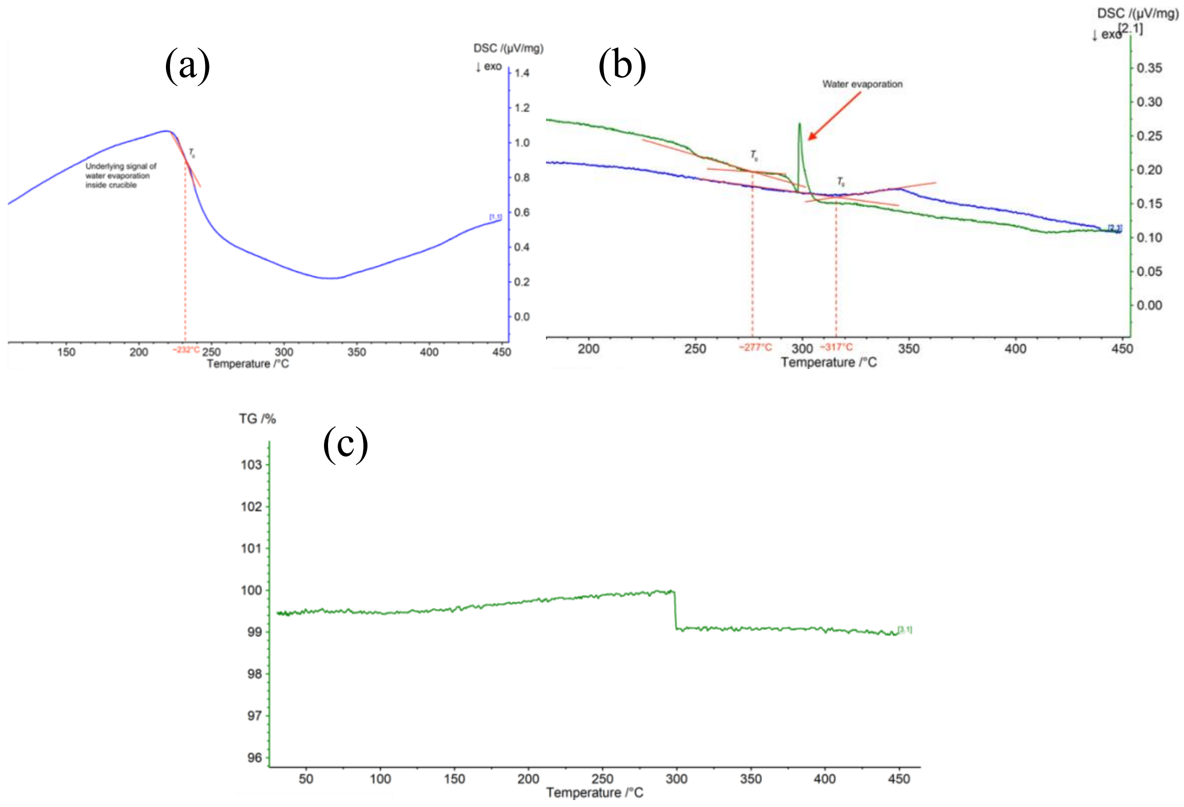

Figure S14. Calorimetry scans for two different glass samples. (a) Glass initially prepared under ambient pressure without water vapor and here scanned in a high-pressure crucible with water corresponding to a maximum pressure of ~55 bar. A glass transition temperature (T_g) is found around 232°C. (b) Glass initially prepared under high pressure with water vapor (~111.1 bar) and here scanned in an Al crucible without water, first yielding a T_g of around 277°C (first upscan, green line) followed by endothermic water release. The second upscan (blue line) shows that T_g relaxes to the ambient pressure value (compare to Fig. 1b in the main text). (c) Thermogravimetric analysis curve for the same sample as in (b) showing a mass loss around 300°C of ~1 wt%.

Figure S15

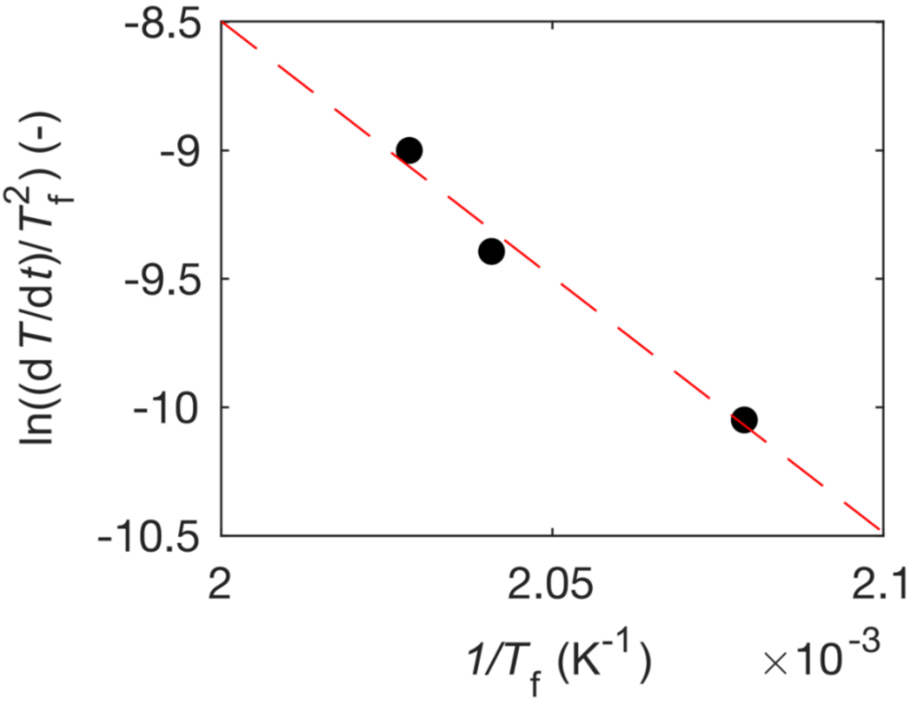

Figure S15. Dependence of the natural logarithm of heating rate (q) and fictive temperature (T_{f}) (i.e., $\ln\left(\frac{q}{T_f^2}\right)$) on the reciprocal of the fictive temperature (T_{f}^{-1}). The slope of linear fit (dashed line) is used to determine the activation energy for viscous flow and subsequently estimate the liquid fragility (m) as described in the Methods section in the main text.

Figure S16

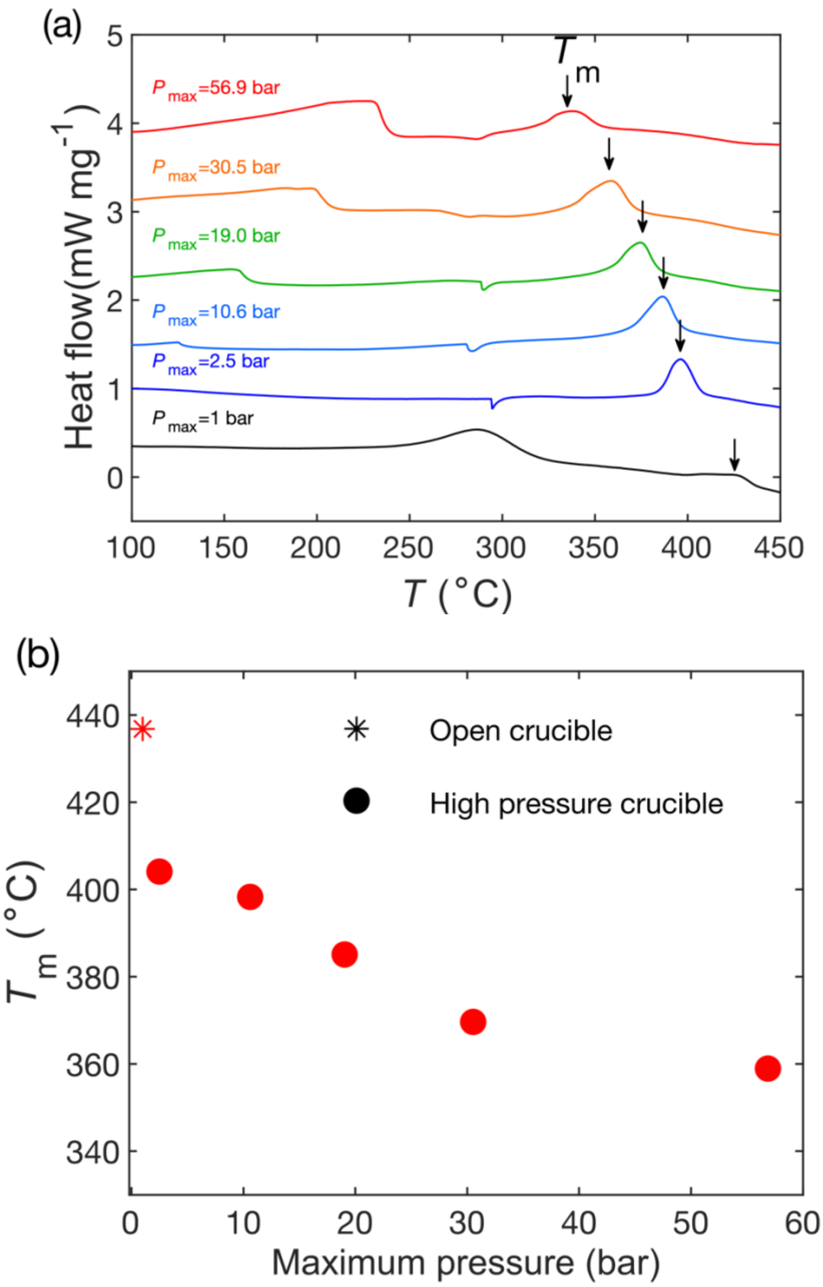

Figure S16. (a) Calorimetry heating scans of un-dried ZIF-62 crystals in an open crucible (black) as well as in sealed 100 μL crucibles where the crystals are mixed with water in varying H_2O:Zn ratios from 0 to 4, corresponding to maximum pressures of 1.0, 2.5, 10.6, 19.0, 30.5, and 56.9 bar, respectively. (b) Suppression of melting temperature (T_m) as a function of the estimated pressure inside the crucible.

Figure S17

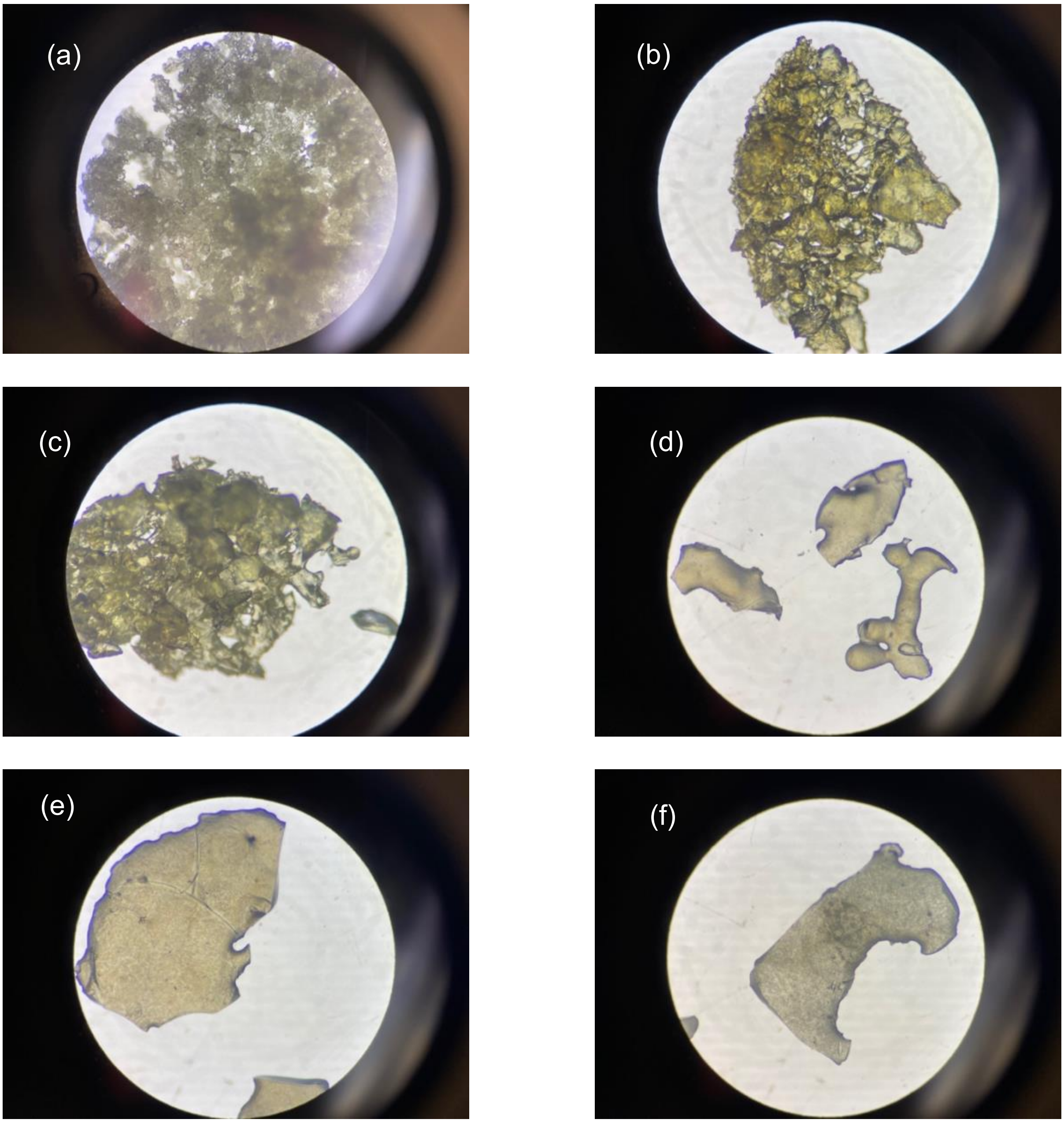

Figure S17. Optical micrographs of formed glasses of undried ZIF-62 glasses (i.e., small amounts of N,N-dimethylformamide (DMF) remains in the ZIF-62 used for the hydrothermal treatment) prepared at (a) 1.0, (b) 2.5, (c) 10.6, (d) 19.0, (e) 30.5, and (f) 56.9 bar, respectively. Samples show significant increase of fluidity upon increasing water pressure. The field of view in each micrograph is approximately 5 mm.

Figure S18

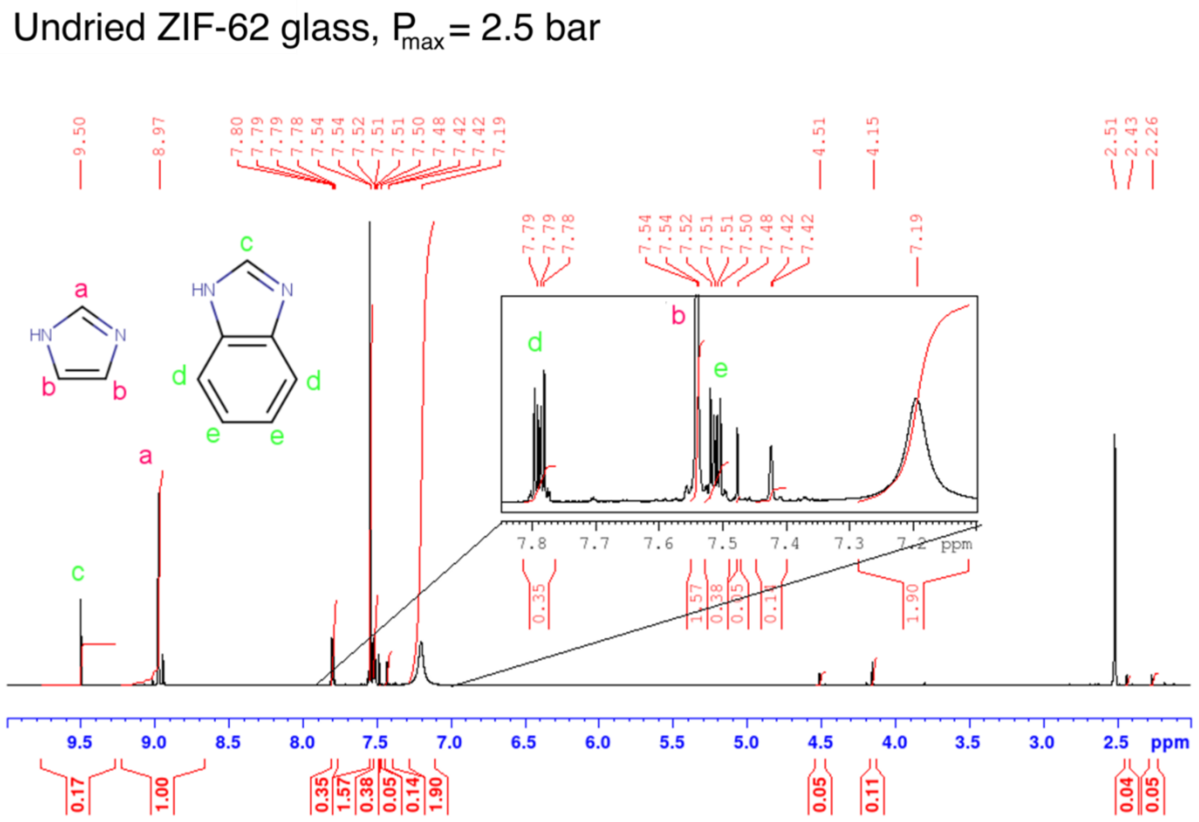

Figure S18. ^{1}H NMR spectrum of undried ZIF-62 glass prepared at a maximum pressure of 2.5 bar (with no water addition). The sample was digested in a mixture of DCl, D_2O, and DMSO-d_6 (see Methods for details). Peak assignments are given for protons in imidazolate (a, b) and benzimidazolate (c, d, e). The spectrum integration is normalized according to the singlet originating from the a-proton in imidazolate (~9 ppm).

Figure S19

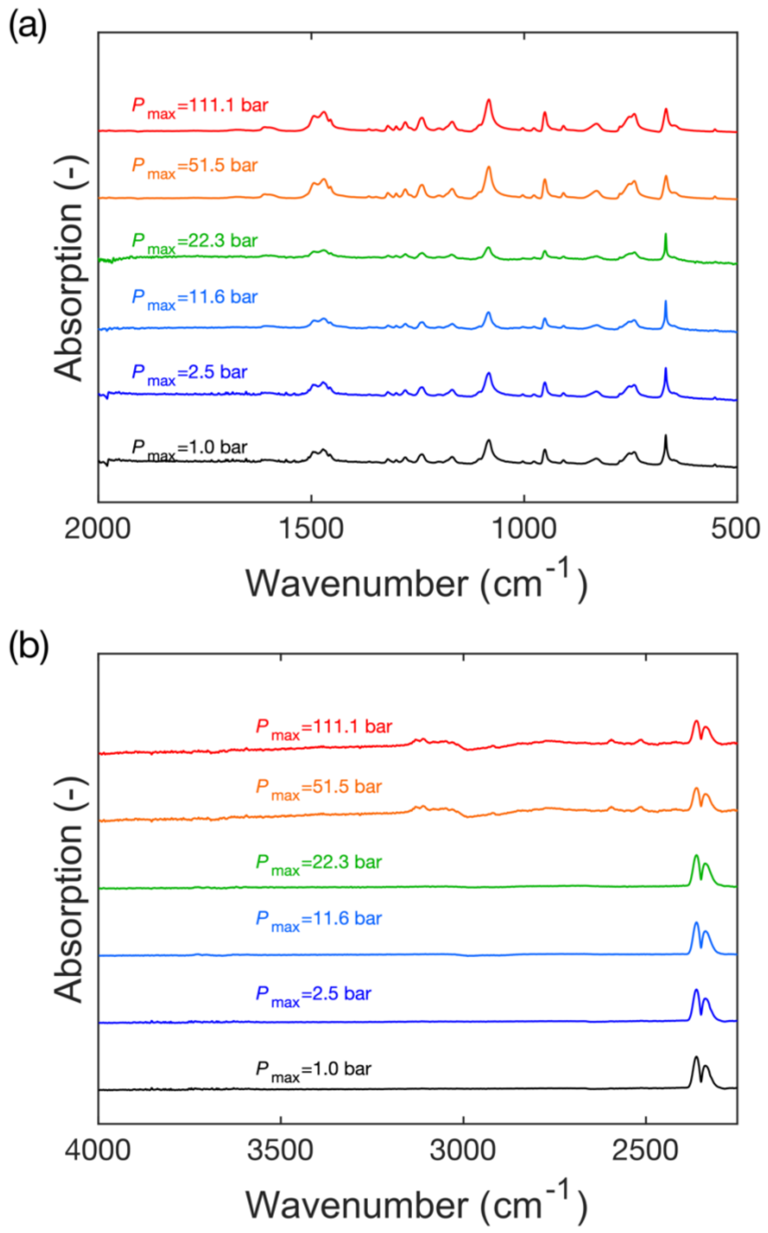

Figure S19. Normalized Fourier transform infrared spectra in (a) the 'fingerprint' region and (b) the high-wavenumber region. In panel (a), all bands seem to not change among the different samples. These bands are signatures of the organic imidazole and imidazolate linkers. In panel (b), a double band is seen at ~2300 cm^{-1} due to insufficient background subtraction of CO_2. However, no major bands are observed in the 3000-3500 cm^{-1} region, which could have been assigned to molecular water or hydroxyl groups.

Figure S20

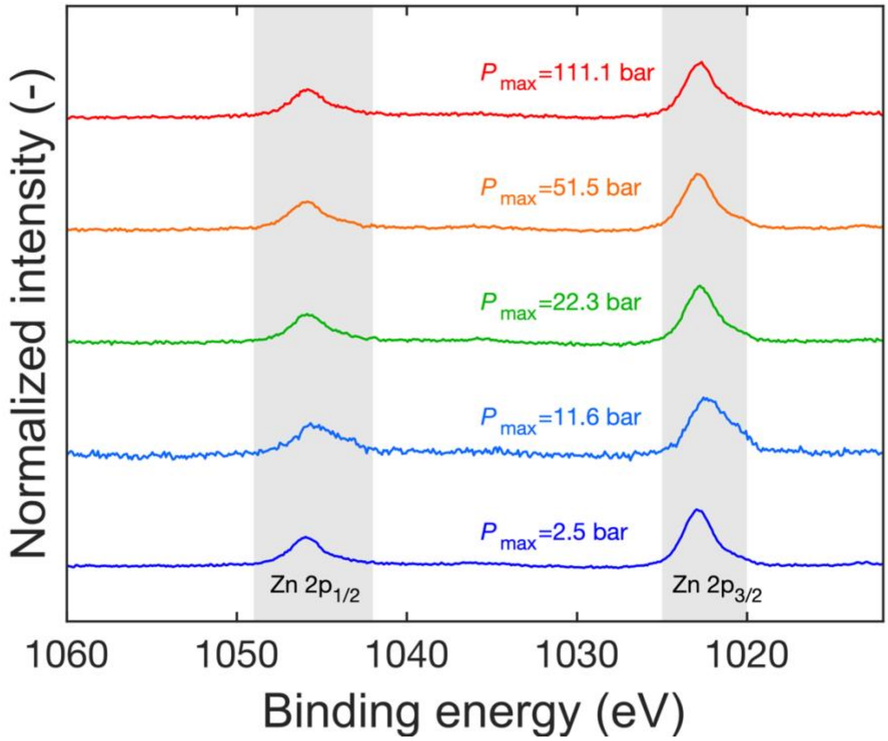

Figure S20. X-ray photoelectron spectra of Zn $2P_{3/2}$ and $2P_{1/2}$ transitions. All samples have been subjected to baseline subtraction. We note that for the P_{max}=11.6 bar sample, slightly more noise is observed compared to that in the remaining samples due to a smaller sample area. Generally, no significant differences in the Zn environments can be identified.

Figure S21

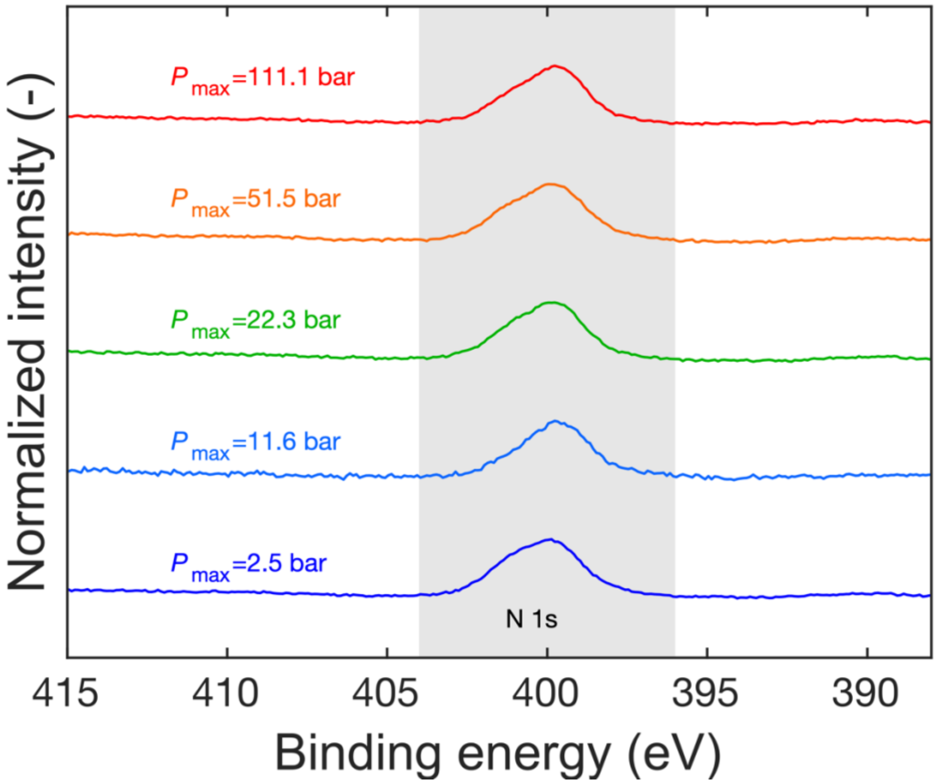

Figure S21. X-ray photoelectron spectra of the N 1s transition. We note that for the P_{max}=11.6 bar sample, slightly more noise is observed compared to that in the remaining samples due to a smaller sample area.

Figure S22

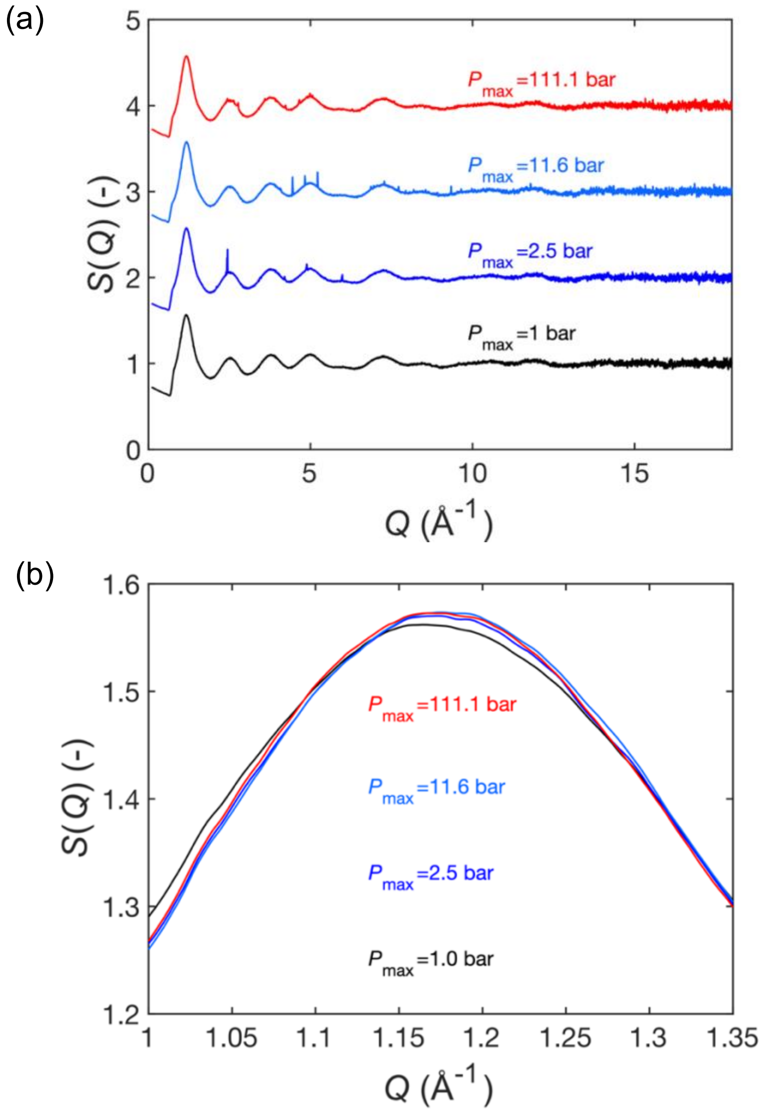

Figure S22. (a) X-ray structure factor $S(Q)$ of the ZIF-62 glass samples prepared at maximum pressures of 1 (black), 2.5 (dark blue), 11.6 (light blue), and 111.1 bar (red). All samples were measured in kapton® capillaries, and we thus performed background and sample container subtraction before normalization. No significant differences are seen among the four recorded sets of scattering data. Small crystalline impurities are seen, however, as noted by the softer X-ray measurements of Figure S9 and the pair distribution function based on the present data (Figure 3d in the main text), these impurities only represent very minor fractions of the total sample volumes. (b) A zoom of the first-sharp diffraction peak in the structure factor $S(Q)$.

Figure S23

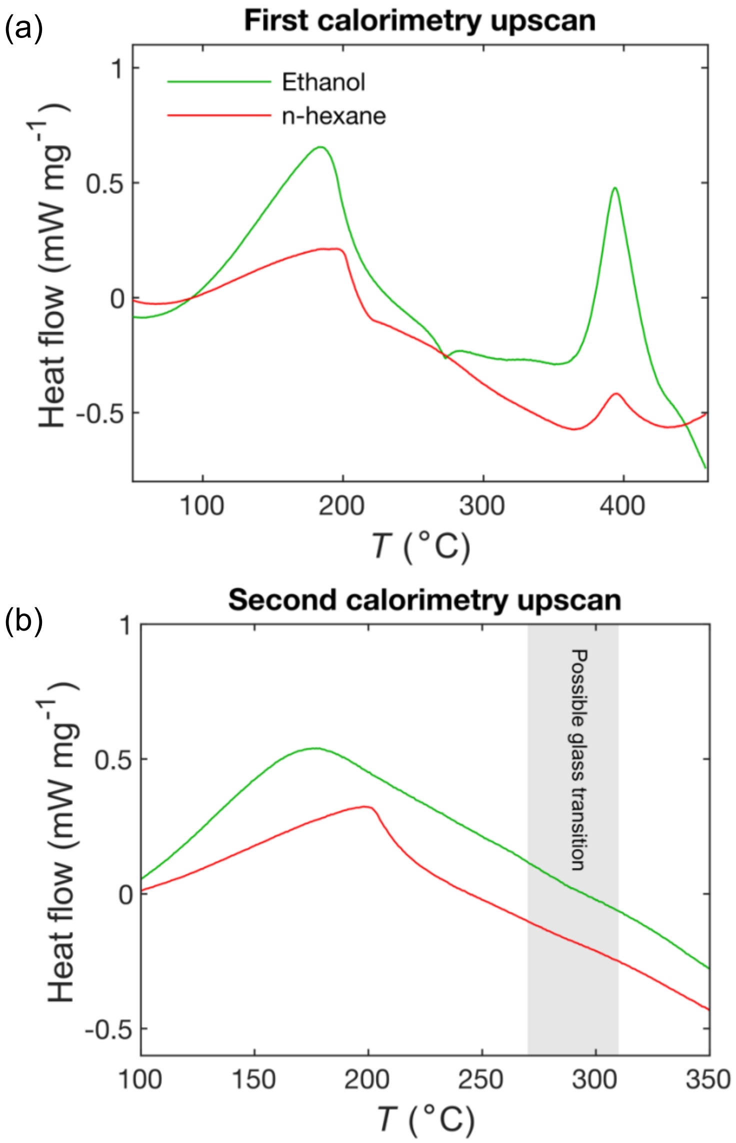

Figure S23. (a) Calorimetry scan of crystalline ZIF-62 mixed with ethanol (green) and *n*-hexane (red). (b) Rescanning of the formed glasses is shown in panel (a). Color coding is the same as in panel (a).

Figure S24

(a)

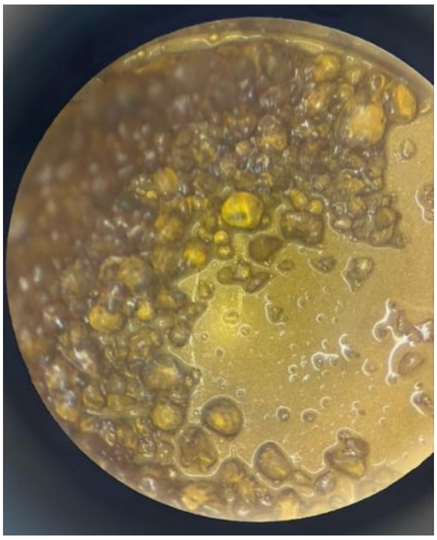

(b)

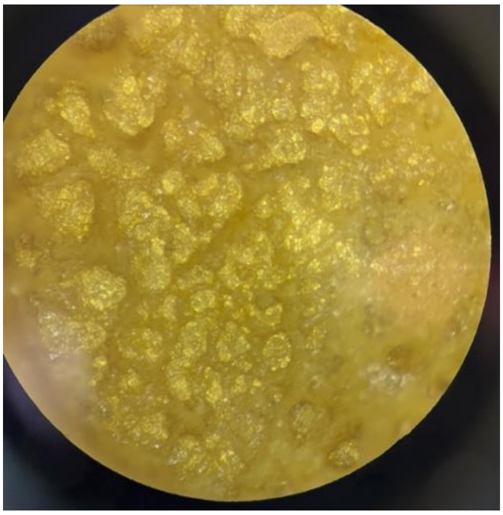

Figure S24. Optical microscopy images of glasses formed by mixing ZIF-62 with (a) ethanol and (b) *n*-hexane. Field of view across the micrograph is ~5 mm. The strong yellow tint is caused by a coating of gold on the crucible walls.

Figure S25

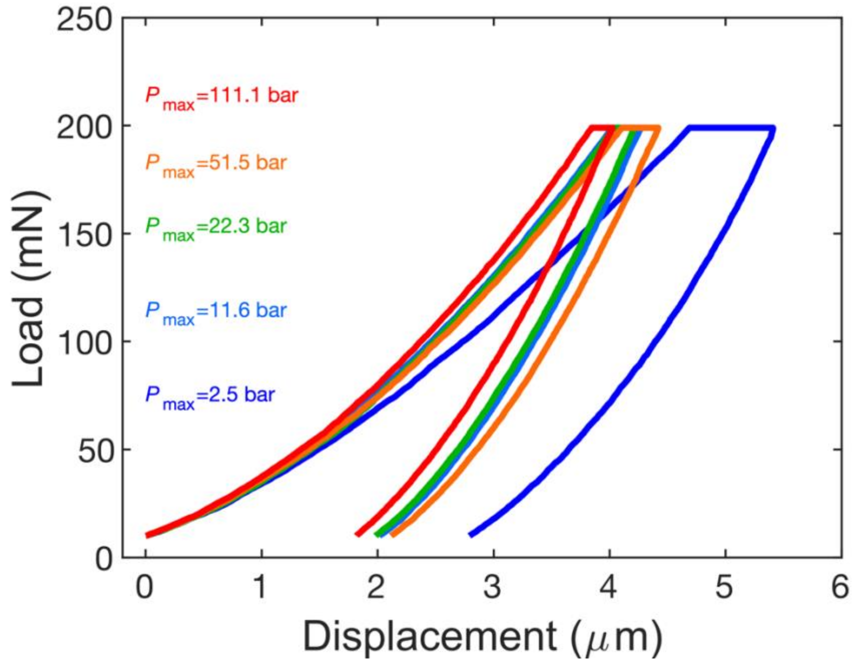

Figure S25. Load-displacement curves from depth-sensing indentation testing, each curve showing the averages of at least eight load-displacements curves for each of the studied ZIF-62 glasses. Measurements were performed using a Vickers indenter. Extracted mechanical properties are shown in Figure 4c-d in the main text.

SUPPORTING REFERENCES

(1) d'Espinose de Lacaillerie, J. B.; Fretigny, C.; Massiot, D. MAS NMR Spectra of Quadrupolar Nuclei in Disordered Solids: The Czjzek Model. *Journal of Magnetic Resonance* **2008**, *192* (2), 244–251. https://doi.org/10.1016/j.jmr.2008.03.001.

(2) Banerjee, R.; Phan, A.; Wang, B.; Knobler, C.; Furukawa, H.; O'Keeffe, M.; Yaghi, O. M.; Rahul Banerjee, Anh Phan, Bo Wang, Carolyn Knobler, H. F.; Michael O'Keeffe, O. M. Y.; O'Keeffe, M.; Yaghi, O. M. High-Throughput Synthesis of Zeolitic Imidazolate Frameworks and Application to CO_2 Capture. *Science (1979)* **2008**, *319* (5865), 939–943. https://doi.org/10.1126/science.1152516.
